# An N-terminal domain specifies developmental control by the SMAX1-LIKE family of transcriptional regulators in *Arabidopsis thaliana*

**DOI:** 10.1073/pnas.2412793122

**Published:** 2025-06-10

**Authors:** Sun Hyun Chang, Wesley J. George, David C. Nelson

**Affiliations:** ^a^Department of Botany and Plant Sciences, University of California, Riverside, CA 92521

**Keywords:** strigolactone, karrikin, regulation, signaling, hormones

## Abstract

SMAX1-LIKE (SMXL) proteins in plants are cellular signaling hubs, many of which are posttranslationally regulated by karrikins from smoke, the plant hormones strigolactones (SLs), and/or cues such as light and nutrients. SMXL proteins control diverse aspects of growth, development, and environmental adaptation in plants through transcriptional corepression and interactions with transcriptional regulator proteins. In flowering plants, the SMXL family comprises four phylogenetic clades with different roles. Functions of the aSMAX1 clade include control of germination and seedling development, while the SMXL78 clade controls shoot architecture. We investigated how SMXL roles are specified in *Arabidopsis thaliana.* Through promoter-swapping experiments, we found that *SMXL7* can partially replicate *SMAX1* function, but *SMAX1* cannot replace *SMXL7*. This implies that the distinct roles of these genes are primarily due to differences in protein sequences rather than expression patterns. To determine which part of SMXL proteins specifies downstream control, we tested a series of protein chimeras and domain deletions of SMAX1 and SMXL7. We found an N-terminal region that is necessary and sufficient to specify control of germination, seedling growth, or axillary branching. We screened 158 transcription factors (TFs) for interactions with SMAX1 and SMXL7 in yeast two-hybrid assays. The N-terminal domain was necessary and/or sufficient for most of the 33 potential protein–protein interactions that were identified for SMAX1. This finding unlocks different ways to engineer plant growth control through cross-wiring SMXL regulatory “input” and developmental “output” domains from different clades and lays a foundation for understanding how functional differences evolved in the SMXL family.

## Significance

Chemicals in smoke known as karrikins and the plant hormones strigolactones (SLs) regulate different aspects of plant growth and development through very similar mechanisms: Evolutionarily related receptors for each pathway work with the same F-box protein to target different members of the SMXL family of transcriptional regulators for degradation. This system provides an opportunity to learn how signaling pathways evolve different roles. We found that the expression pattern of *SMXL* genes is not what determines their unique functions; instead, an N-terminal protein domain specifies SMXL roles. Putatively, this domain allows SMXL proteins to regulate different sets of genes through binding DNA directly and/or selective interactions with transcription factor (TF) partners. This finding enables different strategies to control SMXL-regulated processes in plants.

Plant hormones control growth, development, and responses to the environment through regulation of transcriptional networks (1). Several plant hormones, including auxin, jasmonate, gibberellin, and strigolactone, initiate downstream responses through hormone-triggered polyubiquitination and degradation of transcriptional regulator proteins (2). Strigolactones (SLs), for example, promote protein–protein interactions between an α/β-hydrolase receptor, DWARF14 (D14)/DECREASED APICAL DOMINANCE2 (DAD2); an F-box protein within an SCF (Skp1-Cullin-F-box) E3 ubiquitin ligase complex, MORE AXILLARY GROWTH2 (MAX2)/DWARF3 (D3); and a subset of proteins within the SUPPRESSOR OF MAX2 1 (SMAX1)-LIKE (SMXL)/DWARF(D53) family. The associated SMXL proteins are then polyubiquitinated by SCF^MAX2^ and rapidly destroyed by the 26S proteasome (3–9). This leads to changes in transcriptional activity that initiate downstream growth responses.

SMXL proteins regulate gene expression in two ways: transcriptional corepression and sequestration. First, SMXL proteins recruit TOPLESS (TPL)/TPL-RELATED (TPR) transcriptional corepressor proteins to genomic loci via one or more EAR (Ethylene-responsive element binding factor-associated Amphiphilic Repression) motifs (3, 4, 6, 10, 11). SMXL proteins can bind to DNA directly and also indirectly through protein–protein interactions with transcription factors (TFs) (3–6, 10, 12–16). The EAR motif is important for much, but not all, of SMXL function in *Arabidopsis* (10–12, 14, 17, 18). Second, SMXL protein–protein interactions with transcriptional regulators can affect the stability, regulation, and/or DNA-binding ability of the bound proteins (10, 16, 17, 19–29).

Proteins within the SMXL family have diversified to regulate different developmental processes and to be regulated, in turn, by different signaling mechanisms. In angiosperms, SMXL proteins are grouped into four phylogenetic clades: aSMAX1, SMXL39, aSMXL4, and SMXL78 (15). The aSMAX1-clade proteins, represented by SMAX1 and SMXL2 in *Arabidopsis thaliana* or OsSMAX1 in *Oryza sativa* (rice), regulate seed germination, seedling photomorphogenesis (or in rice, mesocotyl elongation in the dark), root hair density and elongation, drought tolerance, and symbiotic interactions with arbuscular mycorrhizal fungi (20, 30–37). SMAX1 and SMXL2 are targeted for degradation in an SCF^MAX2^-dependent manner by a paralog of D14, KARRIKIN INSENSITIVE2 (KAI2)/HYPOSENSITIVE TO LIGHT (HTL) (18, 37, 38). KAI2 putatively mediates responses to a metabolite of karrikins (KARs), butenolide molecules found in smoke, as well as an undiscovered endogenous compound(s) known as KAI2 ligand (KL) (39). Diversification of KAI2 proteins in some plant lineages has led to selective recognition of different KARs or alternative ligands such as SLs and (–)-germacrene D (40–49). SMAX1 and SMXL2 can also be targeted for degradation by D14–SCF^MAX2^ when SLs are sufficiently abundant (18, 50). SMXL78-clade proteins, represented by SMXL6, SMXL7, and SMXL8 in *Arabidopsis* and D53 in rice, control axillary branching or tillering, secondary growth, leaf elongation, internode elongation, and more (3, 5, 11, 12, 51–54). These proteins are specifically targeted by D14 and not by KAI2 (4, 38, 55). Finally, SMXL3- and aSMXL4-clade proteins, represented by SMXL3, SMXL4, and SMXL5 in *Arabidopsis*, regulate phloem development and anthocyanin abundance (16, 56–59). Unlike other members of the SMXL family, these proteins are not targeted for degradation by SCF^MAX2^, putatively due to loss of a P-loop or Arg-Gly-Lys-Thr (RGKT) motif that stabilizes the KAI2/HTL–SCF^MAX2^–SMAX1 complex (57, 60). In addition to imposing transcriptional regulation on its own, SMXL5, and perhaps its similarly stable homologs, attenuates SL signaling by inhibiting degradation of SMXL7 (16).

The diverse functions of SMXL proteins in plants raise largely unanswered questions of what genes do SMXL proteins regulate and how do they do so? SMXL proteins are distantly related to a ClpB-type heat shock protein, HSP101, that forms hexameric ATPase complexes involved in solubilizing protein aggregates (30, 61). SMXL proteins are composed of a double Clp-N domain (N), a degenerate ATPase domain (D1), a middle region (M), and a second degenerate ATPase domain (D2) (5, 38, 62). Structure–function analyses have revealed roles for several SMXL protein features. The D1M region putatively confers specificity for SMXL interactions with D14 or KAI2 (38). The D2 domain contains the aforementioned RGKT and EAR motifs (3, 4, 6, 10, 11). D2 is necessary for degradation of SMXL proteins but is not sufficient in vivo except when full-length SMXL proteins are also present (38). This may be due to multimeric SMXL complexes that are formed at least in part through interactions at the C-terminus (38). The EAR motif also likely contributes to stabilization of multimeric SMXL complexes (10, 16). Recent cryogenic electron microscopy analysis of a KAI2/HTL–Skp1–MAX2–SMAX1 complex has shown that the D2 and N domains of SMAX1 facilitate protein–protein interactions with KAI2 and/or MAX2 (60). The position and structure of the D1M domains could not be resolved, however, so their function in the protein complex remains unclear.

Several studies have identified direct genomic targets of SMXL proteins or partner proteins that putatively guide the indirect associations of SMXLs with DNA (12, 13, 22, 23, 25, 27, 58, 63, 64). ChIP-seq analysis of SMXL6 revealed 729 candidate target sites in the *Arabidopsis* genome, although there was little overlap with 401 SL-responsive genes (12). Genes directly targeted by SMXL6 include *BRC1*, *SMXL2*, *SMXL6*, *SMXL7*, and *SMXL8* (12). Unexpectedly, SMXL6 and SMAX1 can bind DNA directly; both proteins recognize the motif 5’-ATAACAA-3’ or 5’-TTGTTAT-3’ (12, 13). In rice, D53 interacts with the TFs BRASSINAZOLE RESISTANT 1 (OsBZR1), GROWTH-REGULATING FACTOR 4 (OsGRF4), REDUCED LEAF ANGLE 1 (OsRLA1), DWARF AND LOW TILLERING (OsDLT), and IDEAL PLANT ARCHITECTURE 1 (OsIPA1), as well as the transcriptional regulator SLENDER RICE 1 (OsSLR1), a DELLA protein (21, 23, 25, 27). In *Arabidopsis*, D53-like proteins (SMXL6, SMXL7, and SMXL8) interact with BRI1-EMS SUPPRESSOR 1 (BES1), a homolog of BZR1, and SQUAMOSA PROMOTER BINDING PROTEIN-LIKE 9 (SPL9) and SPL15, homologs of OsIPA1 (23, 65). *Arabidopsis* SMAX1 and SMXL2 interact with DELLA proteins, the TFs PHYTOCHROME-INTERACTING FACTOR 4 (PIF4) and PIF5, and the photoreceptor phytochrome B (13, 17, 20, 22). SMXL3, SMXL4, or SMXL5 interact with OBERON3 (OBE3), a PHD finger protein that putatively recruits chromatin remodeling complexes, and/or the TFs KNOTTED1-LIKE HOMEOBOX GENE 5 (KNAT5) and OVATE FAMILY PROTEIN 1 (OFP1) (58, 63, 64). Therefore, transcriptional regulation by SMXL proteins may arise from a combination of binding specific *cis*-regulatory motifs as well as associating with TFs and transcriptional regulators. Here, we investigated the molecular basis of specificity in transcriptional control by SMXL proteins.

## Results

### *SMAX1* and *SMXL7* are not Interchangeable.

Differential expression of genes in the **a*SMAX1*- and *SMXL78*-clades occurs in many tissue types and developmental stages of *A. thaliana*, although in some cases both types of genes show similar expression (6, 30). For example, **a*SMAX1*-clade transcripts are enriched in seeds and emerging seedlings, while *SMXL78*-clade transcripts are enriched in the roots and apices of older seedlings; however, both types of transcripts are abundant in leaves and floral tissues (*SI Appendix,* Fig. S1) (66).

**Fig. 1.**
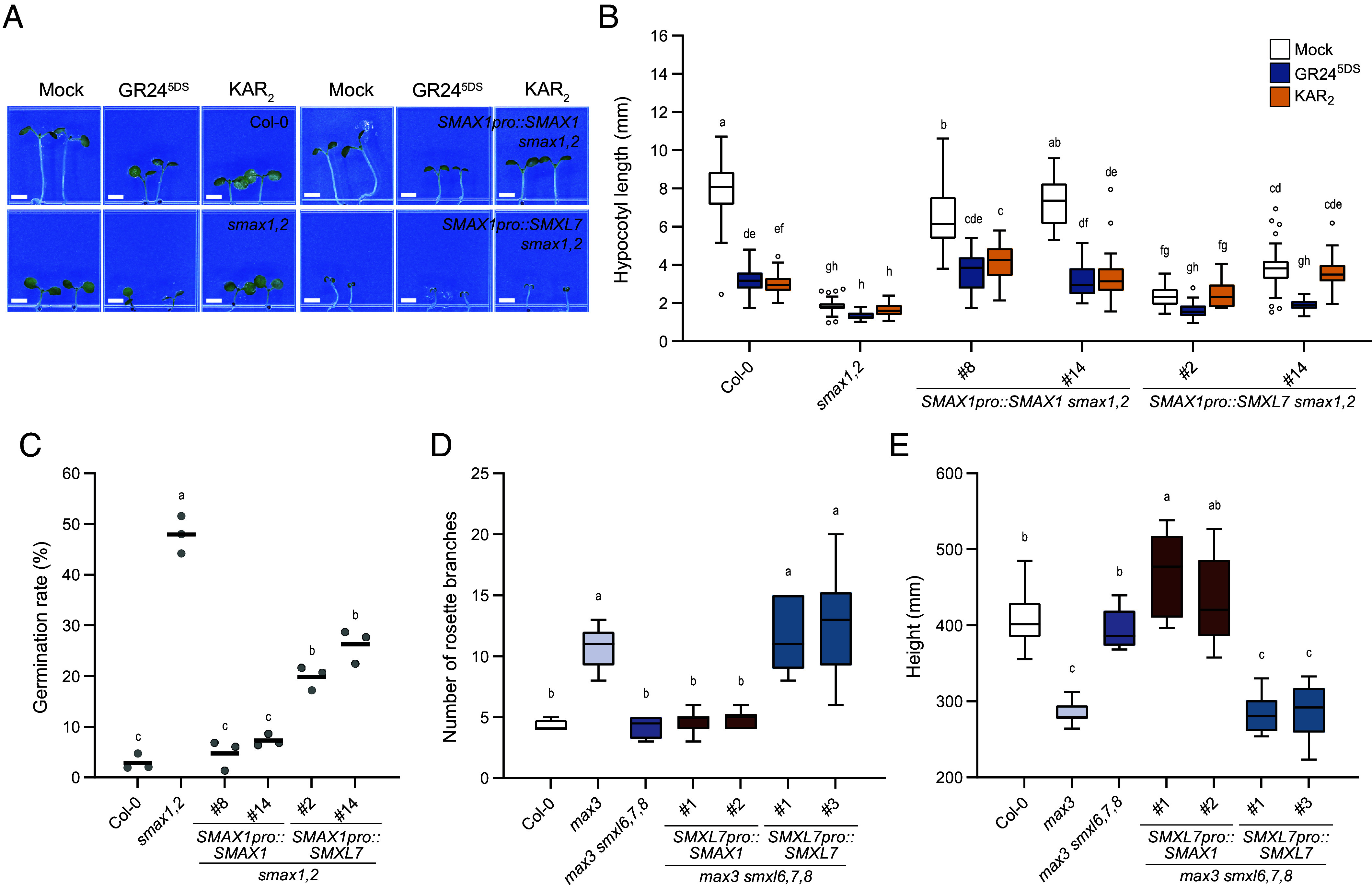
Differences in *SMAX1* and *SMXL7* functions are not due to expression alone. (*A*) Representative image of Col-0, *smax1,2*, *SMAX1pro::SMAX1 smax1,2* #8, and *SMAX1pro::SMXL7 smax1,2* #14 transgenic seedlings grown 6 d in red light. Seedlings were treated with 0.1% acetone, 1 μM KAR_2_, or 1 μM GR24^5DS^. (Scale bar, 2 mm.) (*B*) Measured hypocotyl lengths including the seedlings used in (*A*). (n ≥ 20) (*C*) Germination of transgenic lines expressing *SMAX1* and *SMXL7* under control of the *SMAX1* promoter in *smax1,2* on 3 µM PAC media (n = 3, ≥50 seeds per replicate). (*D*) Rosette branch numbers of 8-wk-old Col-0, *max3*, *max3 smxl6,7,8*, and *SMXL7pro::SMAX1* or *SMXL7pro::SMXL7* in *max3 smxl6,7,8* (n ≥ 9). (*E*) Height of plants in (*D*). Boxplots indicate mean with quartiles and Tukey’s whiskers; open symbols are outlier points that fall beyond the range of the whiskers. Letters indicate groups with significant differences [*P* < 0.05, two-way ANOVA in (*B*), or one-way ANOVA in (*C–E*), followed by Tukey’s multiple comparisons test].

This led us to investigate whether the different roles of the *aSMAX1-* and *SMXL78-*clades in *Arabidopsis* development are due to their expression patterns. We focused on *SMAX1* and *SMXL7* as representative members of each clade because they generally showed the highest expression (*SI Appendix,* Fig. S1). We performed a promoter-swapping experiment in which we tested whether *SMXL7* expressed under the control of a *SMAX1* promoter (*SMAX1pro::SMXL7*) could rescue the *smax1-2 smxl2-1* (hereafter, *smax1,2*) mutant. *SMAX1pro::SMAX1* fully rescued the hypocotyl elongation of *smax1,2* seedlings grown under dim red light. In contrast, *SMAX1pro::SMXL7* partially rescued hypocotyl growth and inhibited cotyledon expansion (Fig. 1 *A* and *B*). This was not a consequence of delayed development, as *SMAX1pro::SMXL7 smax1,2* seedlings showed similar or longer hypocotyl lengths as *SMAX1pro::SMAX1 smax1,2* seedlings at earlier time points of growth under red light (*SI Appendix,* Fig. S2). Similarly, while *SMAX1pro::SMAX1* restored dormancy to *smax1,2* seed, *SMAX1pro::SMXL7* had a significantly weaker effect (Fig. 1*C*). This implies that *SMXL7* shares some function with *SMAX1* but the genes are not equivalent.

Regulation of SMAX1 and SMXL7 in these transgenic seedlings was consistent with prior studies (Fig. 1*B*) (4, 6, 18, 38, 50). Hypocotyl elongation of *SMAX1pro::SMAX1 smax1,2* seedlings was inhibited by KAR_2_ and a synthetic SL, GR24^5DS^, implying KAI2- and D14-induced degradation of SMAX1, respectively. *SMAX1pro::SMXL7 smax1,2* seedlings, however, were responsive to GR24^5DS^ only, consistent with D14-specific targeting of SMXL7 for degradation (Fig. 1*B*). Therefore, even though the *SMXL78* clade has little or no control of hypocotyl elongation in *Arabidopsis* (6, 50), the ability to regulate SMXL7 is present in seedlings.

We then tested the converse situation: Could *SMAX1* replace a *SMXL78*-clade deficiency when expressed under the control of a *SMXL7* promoter? To avoid D14-induced degradation of SMAX1 (18, 50), which might reduce the effectiveness of a *SMXL7pro::SMAX1* transgene, and to maximize the phenotypic differences between rescued and nonrescued lines, we introduced *SMXL7pro::SMAX1* into *max3 smxl6,7,8* rather than *smxl6,7,8*. The *max3 smxl6,7,8* quadruple mutant is SL-deficient, due to the absence of *MORE AXILLARY GROWTH 3* (*MAX3*)/*CAROTENOID CLEAVAGE DIOXYGENASE 7* (*CCD7*), but also has constitutive SL responses due to *smxl6,7,8*. *SMXL7pro::SMXL7* rescued the rosette axillary branching and shoot height phenotypes of *max3 smxl6,7,8* to those seen in the *max3* single mutant. In contrast, *SMXL7pro::SMAX1* did not affect either axillary branch number or plant height (Fig. 1 *D* and *E*). Altogether, these observations demonstrated that the unique functions of *SMAX1* and *SMXL7* are not simply a consequence of their expression patterns. Therefore, SMAX1 and SMXL7 proteins likely regulate distinct developmental processes by regulating different sets of genes, for example, through selective interactions with genomic loci and/or TF partners.

### An N-Terminal “Output” Domain Specifies Developmental Control by SMAX1 and SMXL7.

**Fig. 2.**
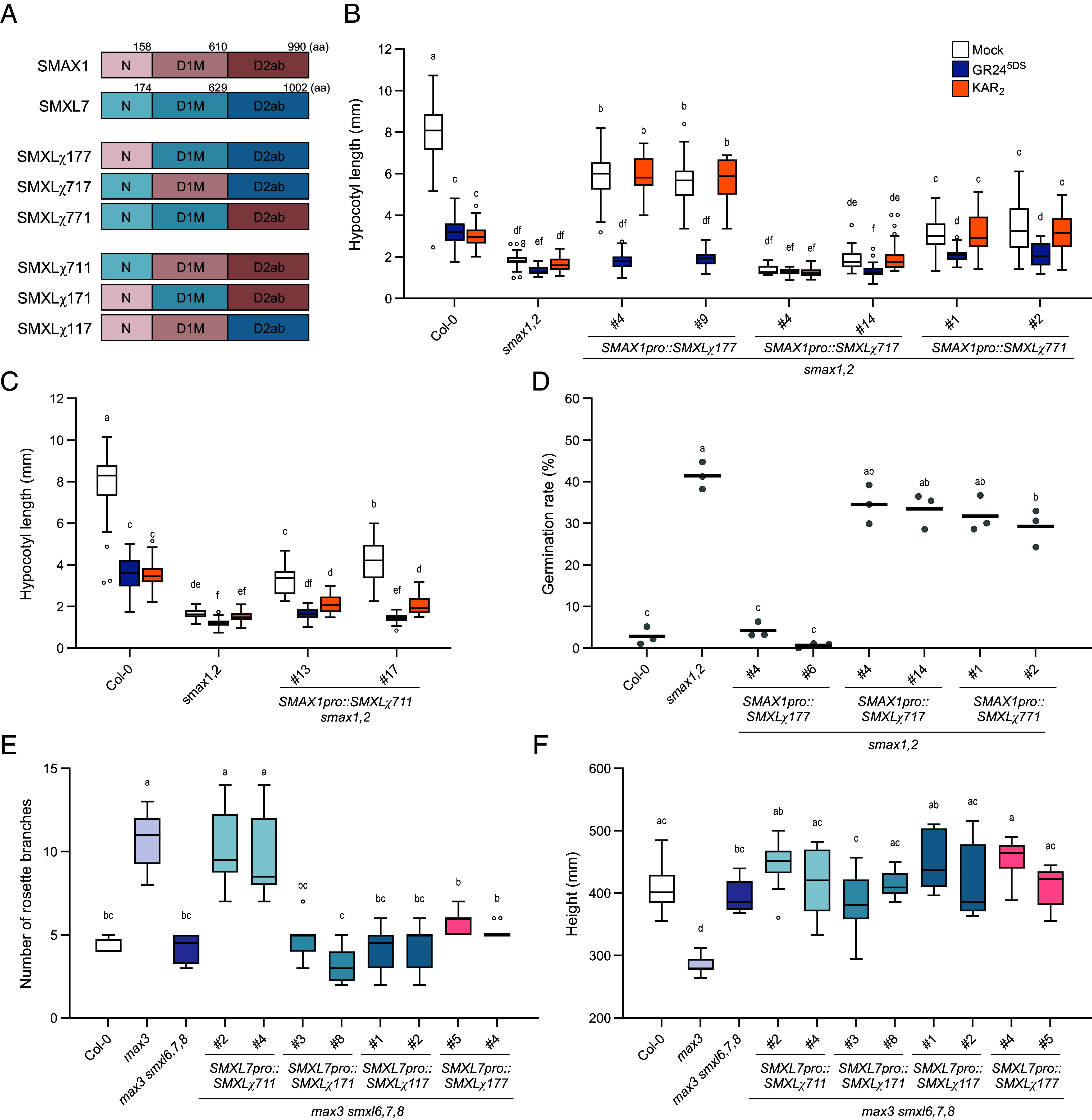
Chimera analysis implicates N domain of SMXL proteins in developmental control (*A*) Schematic of N, D1M, and D2 domain boundaries of SMAX1, SMXL7, and chimeric SMXL proteins. The last amino acid numbers for each domain boundary were marked above the SMAX1 and SMXL7 boundaries. (*B*) Hypocotyl length of red light-grown seedlings treated with 0.1% acetone (mock), 1 μM KAR_2_, or 1 μM GR24^5DS^ (n ≥ 20). (*C*) Hypocotyl length of red light-grown seedlings expressing *SMAX1pro::SMXLχ711* treated with mock, 1 μM KAR_2_, or 1 μM GR24^5DS^ (n ≥ 20). (*D*) Germination of Col-0, *smax1,2*, and transgenic lines expressing *SMXLχ177*, *SMXLχ717*, and *SMXLχ771* under the *SMAX1* promoter in *smax1,2* (n = 3, ≥50 seeds per replicate, 3 µM PAC treatment) (*E*) Rosette axillary branch numbers in 8-wk-old Col-0, *max3*, *max3 smxl6,7,8*, and transgenic lines expressing *SMXLχ711*, *SMXLχ171*, *SMXLχ117,* and *SMXLχ177* under control of the *SMXL7* promoter in *max3 smxl6,7,8* (n ≥ 9). (*F*) Height of the plants in (*E*). Boxplots indicate mean with quartiles and Tukey’s whiskers; open symbols are outlier points that fall beyond the range of the whiskers. Letters indicate groups with significant differences [*P* < 0.05, two-way ANOVA in (*B*), or one-way ANOVA in (*C*–*F*), followed by Tukey’s multiple comparisons test].

To determine which part of SMXL proteins specifies their roles in development, we performed a structure–function analysis. Our first strategy was to swap major domains of SMAX1 and SMXL7 proteins and test the functions of the resulting chimeras (hereafter, SMXL*χ*, where the Greek letter *χ* represents chimera). SMAX1 and SMXL7 proteins putatively have three conserved globular regions connected by less conserved, often intrinsically disordered regions (IDRs) of variable lengths (*SI Appendix,* Figs. S3 and S4*A*) (38, 67, 68). Therefore, to keep the swapped domains in a near-native context within the broader protein structure, we adjusted the previously defined domain boundaries of SMAX1 and SMXL7 to end at nearby, highly conserved residues (*SI Appendix,* Fig. S3). We also considered a prediction of SMAX1 protein structure created by AlphaFold2 (69). This model suggested that our initial C-terminal boundary for the SMAX1 N domain at aa 158 (SMAX1_N158_) is located in the middle of the ninth alpha helix (*SI Appendix,* Fig. S4*B*). This led us to test in some constructs a longer, 210-aa version of the SMAX1 N domain (SMAX1_N210_) that encompasses the predicted globular N-terminal region and part of a putative IDR that follows it. We created a series of reciprocal swaps of the N, D1 and M (D1M), and D2 domains from SMAX1 and SMXL7 (Fig. 2*A*). (The three numbers following SMXL*χ* in each chimera name indicate the source of the N, D1M, and D2 domains, respectively. Unless specified otherwise, the 158-aa version of the SMAX1 N domain was used in the chimeras.) To validate the expression and correct subcellular localization of the SMXL*χ* proteins, we created N-terminal fusions with eYFP and transiently expressed each construct in *Nicotiana benthamiana* leaves. All eYFP-SMXL*χ* proteins produced nuclear-localized fluorescence that was consistent with the localization of wild-type SMAX1 and SMXL7 (*SI Appendix,* Fig. S5).

We then tested whether the SMXL*χ* proteins could rescue *smax1,2* or *max3 smxl6,7,8* when expressed under the control of *SMAX1* or *SMXL7* promoters, respectively. *SMAX1pro::SMXLχ177* mostly rescued the short hypocotyl phenotype of *smax1,2* (Fig. 2*B*). In contrast, *SMAX1pro::SMXLχ771* rescued hypocotyl elongation weakly, similar to *SMAX1pro::SMXL7*, and *SMAX1pro:: SMXLχ717* had no effect (Figs. 1*B* and 2*B*). This suggested that the 158-aa SMAX1 N domain is sufficient to specify control of hypocotyl growth. Furthermore, because *SMXLχ711* had limited ability to rescue *smax1,2* hypocotyl elongation, the N domain may be necessary for SMAX1 function (Fig. 2*C*). In case SMXL7 turnover limits its effectiveness in *smax1,2*, we tested an Arg-Gly-Lys-Thr (RGKT) deletion mutant that is resistant to SL-induced degradation (3–6). Like wild-type SMXL7, SMXL7^ΔRGKT^ recovered hypocotyl elongation of *smax1,2* seedlings only partially (*SI Appendix,* Fig. S6 *A* and *B*). *SMXLχ177*^ΔRGKT^ was more effective at rescuing hypocotyl length, and *SMXLχ1_210_77*^ΔRGKT^ even more so (*SI Appendix,* Fig. S6 *A* and *B*).

Hypocotyl elongation of *SMAX1pro::SMXLχ177* and *SMAX1pro::SMXLχ771 smax1,2* seedlings was inhibited by GR24^5DS^ treatment, but not by KAR_2_ (Fig. 2*B*). This suggests that these chimeric proteins, which share the D1M domain from SMXL7, are targeted for degradation by D14 but not KAI2. It provides further evidence that the D1M domain specifies SMXL interactions with D14 or KAI2 receptors (38) and also demonstrates cross-wiring of the KAR and SL signaling systems.

Unexpectedly, we also observed hypocotyl elongation responses to GR24^5DS^ in *SMXL7^ΔRGKT^*, *SMXLχ177^ΔRGKT^*, and *SMXLχ1_210_77^ΔRGKT^ smax1,2* lines (*SI Appendix,* Fig. S6 *A* and *B*). While this might indicate that the RGKT deletion does not confer SL-induced degradation resistance to SMXL7, similar mutations of *Arabidopsis* SMXL6 and SMXL7 have shown a stabilizing effect (4, 11). In addition, in the *max3 smxl6,7,8* background, SMXL7^ΔRGKT^ produced significantly more rosette axillary branches compared to *max3* while SMXL7 did not, suggesting it has stronger rescuing activity (*SI Appendix,* Fig. S7*C*). At this time, we cannot explain the genetic background-specific effect of *SMXL7^ΔRGKT^*. Perhaps a lack of competition for SCF^MAX2^ interaction by SMAX1 and SMXL2 in the *smax1,2* background enables better targeting of SMXL7^ΔRGKT^, which putatively would have weakened interactions within a D14–SCF^MAX2^ complex (60).

We found further support for the importance of the SMXL N domain in developmental control specificity in germination assays of the *SMXLχ smax1,2* transgenic lines. *SMAX1pro::SMXLχ177* restored seed dormancy to *smax1,2,* but *SMAX1pro::SMXLχ717* and *SMAX1pro::SMXLχ771* did not affect germination significantly (Fig. 2*D*). Likewise, *SMXLχ177*^ΔRGKT^ and *SMXLχ1_210_77*^ΔRGKT^ rescued seed dormancy to a greater degree than *SMXL7*^ΔRGKT^ (*SI Appendix,* Fig. S6 *A* and *C*).

We next investigated whether the N domain of SMXL7 specifies control of axillary branching and shoot height (Fig. 2 *E* and *F*). We developed chimeric proteins with SMAX1 to test a 174-aa SMXL7 N domain, which ends at an equivalent position to aa 158 in SMAX1 (*SI Appendix,* Fig. S3). *SMXL7pro::SMXLχ711* restored the axillary branching of *max3 smxl6,7,8* plants to the level of *max3*, as we had observed for wild-type *SMXL7* but not *SMAX1* transgenes (Figs. 1*D* and 2*E*). In contrast, *SMXL7pro:: SMXLχ171* and *SMXL7pro::SMXLχ117* did not affect axillary branching (Fig. 2*E*). This suggests that the 174-aa N domain of SMXL7 is sufficient to specify control of axillary branching. *SMXL7pro::SMXLχ177* also did not affect the axillary branching phenotype of *max3 smxl6,7,8* (Fig. 2*E*), implying that the SMXL7 N domain is necessary for axillary branching control. Interestingly, none of the chimeras affected the height of *max3 smxl6,7,8* plants (Fig. 2*F*). Therefore, regions of SMXL7 protein in addition to the N domain may be required to control shoot height. As well as having increased axillary branching from the rosette (i.e., primary branches), *max3* has excess secondary branching from cauline nodes (70). We found that *SMXL7pro::SMXLχ711* did not rescue secondary branching (*SI Appendix,* Fig. S7 *C* and *D*). In addition, the rosette morphology of 3-wk-old *SMXL7pro:: SMXLχ711 max3 smxl6,7,8* plants was similar to *max3 smxl6,7,8* and unlike *SMXL7pro::SMXL7 max3 smxl6,7,8* plants (*SI Appendix,* Fig. S7*E*). Therefore, the SMXL7 N domain within a SMAX1 protein context is only sufficient to specify regulation of rosette axillary branching.

To validate the role of the N domain in specifying output control by SMXL proteins, we examined the expression of several KAR/KL- and SL-responsive marker genes known to be regulated by SMAX1 and SMXL7 with RT-qPCR (12, 17, 30, 71–75). In red light-grown seedlings, we observed that *SMXLχ177* caused downregulation of *D14-LIKE 2* (*DLK2*)*, KAR-UPREGULATED F-BOX 1* (*KUF1*), and *ETHYLENE RESPONSE FACTOR 61* (*ERF61*) transcripts similarly to *SMAX1* and to a greater degree than *SMXL7* (*SI Appendix,* Fig. S8*A*). This aligns with the partial effectiveness of *SMXL7* in rescuing *smax1,2* hypocotyl growth and germination compared to *SMAX1* and *SMXLχ177* (Figs. 1 *A* and *B* and 2*B* and *SI Appendix,* Fig. S8*A**)*. However, some KAR/KL marker genes showed unexpected expression patterns. *B-BOX DOMAIN PROTEIN 20* (*BBX20*)/*SALT TOLERANCE HOMOLOG 7* (*STH7*) expression was upregulated in *smax1,2* compared to Col-0, as previously reported (33, 76). Surprisingly, *SMAX1* and *SMXL7* further enhanced *BBX20* transcript abundance, but *SMXLχ177* did not (*SI Appendix*, Fig. S8*A*). Additionally, expression of *INDOLE-3-ACETIC ACID INDUCIBLE 29* (*IAA29*), which is downregulated in *smax1,2*, was recovered by *SMAX1* and *SMXL7* but not by *SMXLχ177* (*SI Appendix,* Fig. S8*A*). Thus, the *SMXLχ177* chimera reconstituted some, but not all, gene regulation by *SMAX1*.

In the aerial tissues (rosette leaves and shoots) of 5-wk-old transgenic *max3 smxl6,7,8* plants, *SMXLχ711* reduced the transcript abundance of *BRANCHED 1* (*BRC1*)/*TEOSINTE BRANCHED1/CYCLOIDEA/PROLIFERATING CELL FACTOR 18* (*TCP18*) similarly to *SMXL7*, whereas *SMAX1* did not. *SMXL7* also reduced *TCP1* expression in *max3 smxl6,7,8* plants while *SMAX1* had the opposite effect. The effect of *SMXLχ711* on expression of *TCP1* was clearly different from *SMAX1* and not significantly different from either *SMXL7* or the nontransgenic *max3 smxl6,7,8* background. While these results suggest the chimeric protein has *SMXL7*-like activity, it might also occur if the protein is nonfunctional (*SI Appendix,* Fig. S8*B*). Finally, *SMXL7* suppressed the expression of *PRODUCTION OF ANTHOCYANIN PIGMENT 1* (*PAP1*) in *max3 smxl6,7,8,* while *SMAX1* did not. *SMXLχ711* showed intermediate effects that were not significantly different from either *SMAX1* or *SMXL7*.

Collectively, these results indicate that the N domains of SMAX1 and SMXL7 play important, although not exclusive, roles in specifying downstream developmental and transcriptional signaling outputs. Other domains may also contribute to the distinct functions of these proteins, in particular for SMXL7 based upon the limited functionality of the SMXL*χ*711 chimera in the control of shoot architecture. An in-depth transcriptomic analysis will be required to determine which genes are under SMXL N domain-dependent control and which are not.

### SMAX1_N_ Is Necessary and Sufficient for Regulating Early Development.

**Fig. 3.**
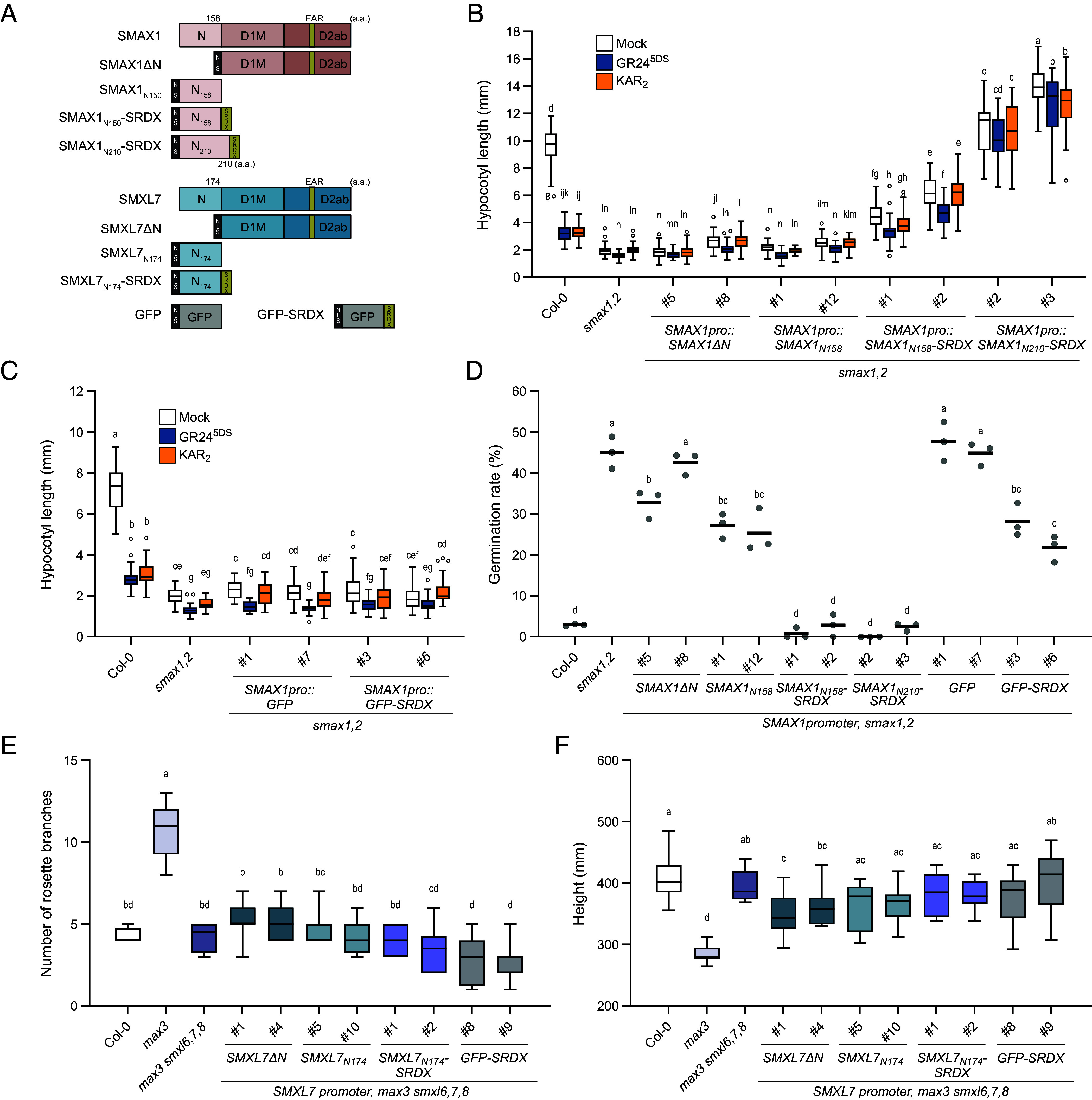
SMAX1 N domain specifies control of *Arabidopsis* germination and seedling growth (*A*) Schematic representation of truncated SMAX1 and SMXL7 proteins. GFP and GFP-SRDX were used as negative controls. Black, SV40 NLS added to the N termini of truncated SMXL proteins. Olive green, EAR or SRDX motifs. (*B*) Hypocotyl length of red light-grown seedlings treated with 0.1% acetone (mock), 1 μM KAR_2_, or 1 μM GR24^5DS^ (n ≥ 20) of Col-0, *smax1,2*, and transgenic lines expressing *SMAX1ΔN, SMAX1_N158_, SMAX1_N158_-SRDX,* and *SMAX1_N210_-SRDX* under control of the *SMAX1* promoter in *smax1,2*. (*C*) Hypocotyl length of red light-grown seedlings treated with mock, 1 μM KAR_2_, or 1 μM GR24^5DS^ (n ≥ 20) of Col-0, *smax1,2*, and transgenic lines expressing *GFP* and *GFP-SRDX* under control of the *SMAX1* promoter in *smax1,2*. (*D*) Germination rates of seeds under (n = 3, ≥50 per replicate, 3 µM PAC treatment). (*E*) Rosette axillary branch numbers of 8-wk-old Col-0, *max3*, *max3 smxl6,7,8*, and transgenic lines expressing *SMXL7ΔN, SMXL7_N174_,* and *SMXL7_N174_-SRDX* under *SMXL7* promoter (n ≥ 9). (*F*) height of plants in (*E*). Boxplots indicate mean with quartiles and Tukey’s whiskers; open symbols are outlier points that fall beyond the range of the whiskers. Letters indicate groups with significant differences [*P*<0.05, two-way ANOVA in (*B* and *C*), or one-way ANOVA in (*D–F*), followed by Tukey’s multiple comparisons test].

To further investigate the role of the N domain in developmental regulation by SMAX1 and SMXL7, we performed a domain deletion analysis. We fused an N-terminal nuclear localization signal (NLS) from simian virus 40 (SV40) to truncated SMAX1 and SMXL7 proteins in order to maintain the correct subcellular localization (Fig. 3*A*). *SMAX1pro::SMAX1ΔN* failed to rescue *smax1,2* hypocotyl elongation or seed dormancy (Fig. 3 *B* and *D*). Similarly, *SMXL7pro::SMXL7ΔN* did not rescue the axillary branching or shoot height phenotypes of *max3 smxl6,7,8* (Fig. 3 *E* and *F*). Therefore, the N domain is necessary for SMAX1 and SMXL7 functions.

We also tested SMAX1_N158_ alone and found that it had no effect on *smax1,2* hypocotyl growth or germination (Fig. 3 *B* and *D*). This was not surprising, as many SMXL functions are dependent on a C-terminal EAR motif(s) that facilitates interactions with TPL/TPR transcriptional corepressors (3, 4, 6, 10, 11, 16). To better mimic SMAX1 function, we fused SRDX, a transcriptional repression domain derived from the EAR motif sequence of SUPERMAN/FLORAL DEFECTIVE 10 (77), to the C-terminus of SMAX1_N158_. *SMAX1pro::SMAX1_N158_-SRDX* moderately recovered hypocotyl elongation of *smax1,2* and restored seed dormancy (Fig. 3 *B* and *D*). A similar fusion with the longer, 210-aa version of the SMAX1 N domain was more effective. *SMAX1_N210_-SRDX* robustly rescued *smax1,2*, causing hypocotyl elongation to exceed that of wild-type Col-0 (Fig. 3*B*). This was not a consequence of SRDX alone, as *SMAX1pro::GFP-SRDX* had no effect on hypocotyl elongation of *smax1,2* and only affected seed germination weakly (Fig. 3 *C* and *D*). These results demonstrate that SMAX1_N_, in particular the 210-aa version, is sufficient to specify regulation of germination and seedling growth when fused to SRDX. We also observed that SMAX1_N210_-SRDX suppressed expression of *DLK2, KUF1, ERF61*, and *BBX20,* and increased *IAA29* expression in *smax1,2* seedlings. This supports the sufficiency of the N domain to specify gene regulation by SMAX1 (*SI Appendix,* Fig. S8*A*).

Notably, SMAX1_N210_-SRDX appeared to reconstitute the function of full-length SMAX1 but not its regulation by SCF^MAX2^, as *SMAX1_N210_-SRDX smax1,2* seedlings were insensitive to KAR_2_ and GR24^5DS^ treatments (Fig. 3*B*). We did not test the effects of SMAX1_N210_ without an SRDX fusion; therefore, we cannot distinguish which functions of this domain may be EAR motif-independent (i.e. action via sequestration of specific transcriptional regulator(s) rather than TPL/TPR-mediated transcriptional corepression). However, it was previously shown that SMAX1 regulation of *ERF61* and *IAA29* is EAR motif-independent, while regulation of *KUF1* and *BBX20* is EAR motif-dependent (17).

We similarly tested the necessity and sufficiency of SMXL7_N174_ for regulating SL responses. *SMXL7pro::SMXL7ΔN* did not rescue the axillary branching or shoot height phenotypes of *max3 smxl6,7,8*, supporting the necessity of the N domain for these functions (Fig. 3 *E* and *F*). However, *SMXL7_N174_* and *SMXL7_N174_-SRDX* also failed to rescue *max3 smxl6,7,8* even though SMXL7_N174_ was sufficient to confer axillary branching control to the SMXL*χ*711 chimera (Fig. 2*E*). A different-length N domain may be required to recapitulate SMXL7 function in an SRDX fusion or other domains may also be required to coordinate gene regulation.

### The N Domain Putatively Mediates SMXL Interactions with Many TFs.

SMXL6 and SMAX1 have been reported to bind to the same DNA motif (12, 13). This implies that additional factors are required to achieve distinct developmental outputs. We reasoned that SMAX1 may control different gene regulatory networks than SMXL7 through differential interactions with TF protein partners. This led us to screen for potential TF partners of SMAX1 and SMXL7, simultaneously examining the importance of the N domain for such interactions.

**Fig. 4.**
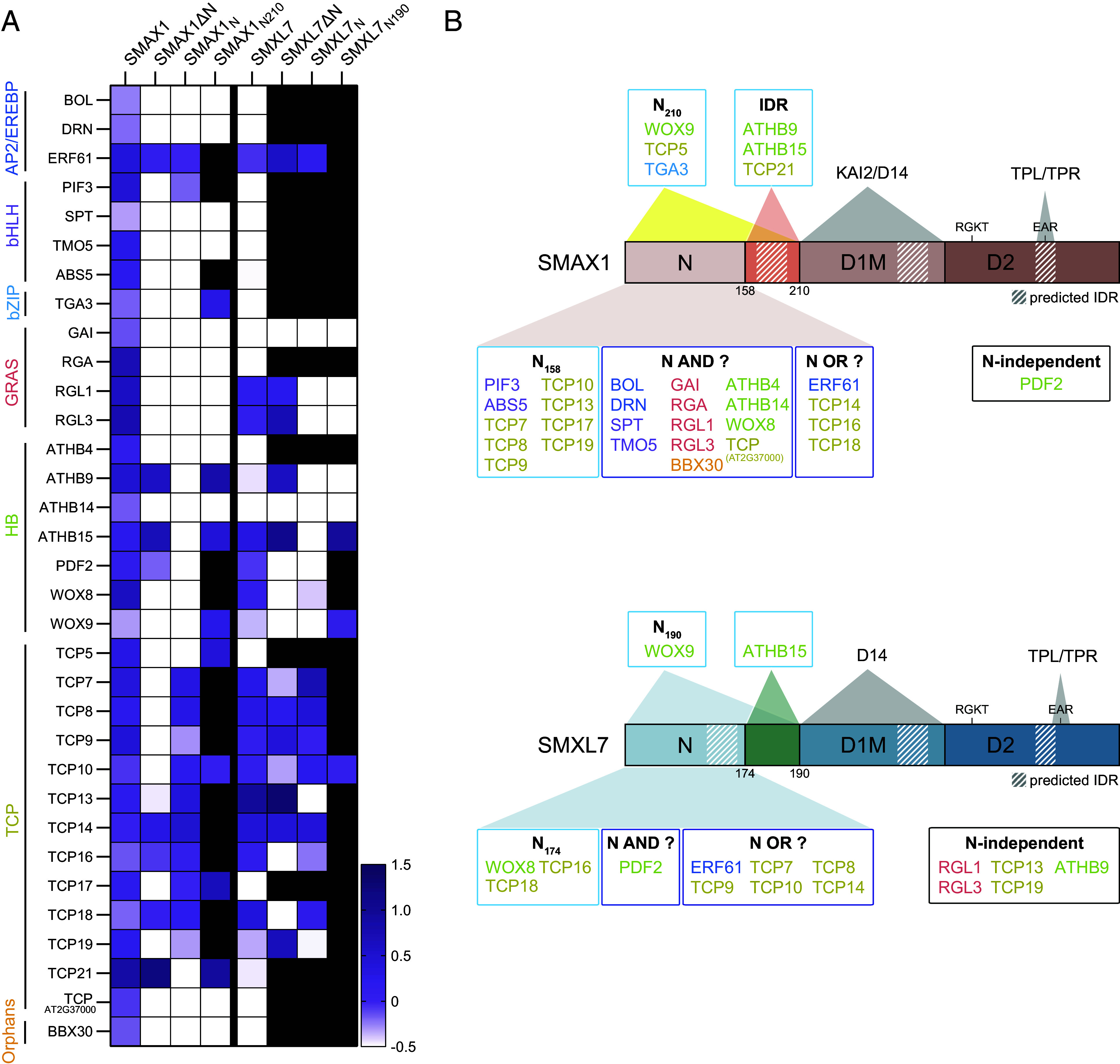
SMAX1 N domain is involved in most of the potential interactions with transcription factors (TFs). (*A*) Heatmap summarizing positive Y2H interactions between full-length and truncated SMAX1 or SMXL7 proteins with various TFs. Interaction level was quantified by comparing yeast growth on –LW and –LWH media and presented on a log_2_ scale. Interactions with values above −0.5 (purple) were considered positive, while below −0.5 (white) were considered noninteracting. Black boxes indicate untested combinations. (*B*) Schematic of the TFs that interact with the N-terminal domain of SMAX1 (*Top*) or SMXL7 (*Bottom*). TFs are grouped based on their interaction requirements with the SMAX1/SMXL7 N domain: “N_158_” or “N_174_", TFs for which the N domain is both necessary and sufficient for interactions; “N AND ?,” TFs that require the N domain and an additional SMXL region(s) for interaction; “N OR ?,” TFs for which the N domain is sufficient but not necessary for interaction. Hatched boxes, three longest predicted intrinsically disordered regions (IDRs) based on *SI Appendix*, Fig. S4*A*. TFs belonging to the same family are depicted in the same color in (*A* and *B*).

We conducted yeast two-hybrid (Y2H) assays with 158 transcriptional regulators from an *A. thaliana* TF library (78). The following criteria aided our selection of candidate TFs (*SI Appendix,* Table S1): 1) known physical and/or genetic interactions with aSMAX1- or SMXL78-clade proteins, 2) putative direct targets of SMXL6, 3) differential expression after GR24 treatment, and 4) association with seed development/germination, photomorphogenesis, root hair development, or leaf morphology (12, 79). We were most interested in identifying potential interactions between candidate TFs and SMAX1, which has been less characterized, but we also tested for interactions with SMXL7 (Fig. 4 and *SI Appendix,* Fig. S9–S11 and Table S2). The respective N domains (SMAX1_N158_ and/or SMXL7_N174_) or SMXL proteins lacking the N domain (SMAX1ΔN and/or SMXL7ΔN) were also tested to determine the basis of any positive Y2H interactions. If a TF interacted with a full-length SMXL protein but not the N domain alone, we tested whether it could interact with a longer version of the N domain (SMAX1_N210_ and/or SMXL7_N190_), as SMAX1_N210_-SRDX had proven more effective than SMAX1_N158_-SRDX in transgenic plants.

We identified 33 TFs that showed positive Y2H interactions with full-length SMAX1. The majority of these interactions (25 of 33) required the 158-aa SMAX1 N domain (Fig. 4 and *SI Appendix,* Fig. S9−S11). The 33 potential SMAX1–TF interactions could be divided into 1) 12 for which the short or long SMAX1 N domain was necessary and sufficient (“N_158_” or “N_210_”); 2) 13 that required the N domain and another part of the SMAX1 protein (“N AND ?”), as the N domains were necessary but not sufficient; 3) four that could interact with either the N domain or another part of the SMAX1 protein (“N OR ?”), as the N domains were sufficient but not necessary; 4) three that likely involved the IDR that distinguishes the long and short versions of the N domain (“IDR”); and 5) one that probably did not involve the N domain, as it interacted with SMAX1ΔN but none of the N domains (Fig. 4 and *SI Appendix*, Fig. S9).

We found that 17 of the 33 SMAX1-interacting TFs also interacted with SMXL7 or its truncated derivatives, and we did not observe any SMXL7-specific interactions (Fig. 4). In contrast to SMAX1, a smaller proportion of these interactions (5 of 17) required the 174-aa SMXL7 N domain (Fig. 4 and *SI Appendix,* Fig. S9−S11). The 17 potential SMXL7–TF interactions could be divided into 1) four for which a 174-aa or longer, 190-aa SMXL7 N domain was necessary and sufficient (“N_174_” or “N_190_”); 2) one that required the N domain and another part of the SMXL7 protein (“N AND ?”), as the N domains were necessary but not sufficient; 3) six that could interact with either the N domain or another part of the SMXL7 protein (“N OR ?”), as the N domains were sufficient but not necessary; 4) one that could interact with the region that distinguishes the long and short versions of the N domain; and 5) five that did not involve the N domain, as it interacted with SMXL7ΔN but none of the N domains (Fig. 4).

Altogether, these observations support the importance of the N domain in transcriptional control by SMAX1 and, to a lesser degree, SMXL7. We have identified a set of TFs that might be involved in developmental regulation by SMAX1 or SMXL7. Some of the differential Y2H interactions we observed with TFs may explain the unique roles of SMAX1 and SMXL7 in plant development (Fig. 4 and *SI Appendix,* Fig. S9−S11). However, these candidate protein–protein interactions will require further validation in vivo.

## Discussion

Initial studies of *SMXL* genes in *Arabidopsis* showed that their developmental roles often corresponded with the tissues in which their expression was enriched. For example, *SMAX1* has high expression in seeds and seedlings, *SMXL7* is highly expressed in axillary branches (*SI Appendix,* Fig. S1), and *SMXL3*, *SMXL4*, and *SMXL5* are highly expressed in phloem (6, 30, 57). Here, we have found that the different expression patterns of *SMXL* genes are not sufficient to explain their varied functions. *SMXL7* under control of the *SMAX1* promoter is not as effective as *SMAX1* at rescuing *smax1,2* seedlings. Neither is *SMAX1* under control of the *SMXL7* promoter able to rescue the *SMXL* deficiency of *max3 smxl6,7,8* plants. Furthermore, *SMXL7* expressed under control of the *SMAX1* promoter appears to have comparable effectiveness in the control of rosette morphology, shoot branching, inflorescence height, and downstream gene expression as *SMXL7pro:: SMXL7* or the hypermorphic *SMXL7pro::SMXL7^ΔRGKT^* transgenes, suggesting that a *SMXL7*-specific expression pattern may not be critical (*SI Appendix,* Figs. S7 *A*, *B,* and *E* and S8*B*). Similarly, misexpression of *SMXL5* under the control of *SMAX1* or *SMXL7* promoters does not rescue *smax1,2* or *smxl6,7,8*, and *SMXL5* promoter-driven expression of *SMAX1* and *SMXL7* only rescues *smxl4,5* partially (16, 57). Altogether, this implies that SMXL proteins are not interchangeable and that unique sequence features underlie their different functions.

This study implicates the N domain of SMXL proteins as a major determinant of SMXL roles in plant growth and development, in particular for SMAX1. This conclusion is supported by a prior observation that the N domain of SMAX1 mediates interactions with phytochrome B (20). In addition, the first exon of SMXL4/AtHSPR, which encodes the N domain and part of the D1 domain, is necessary and sufficient for protein–protein interactions with the TFs KNAT5 and OFP1 (64). It remains to be seen whether the N domain is responsible for direct DNA-binding by SMXL proteins or only interactions with TFs. Because the SMAX1 N domain is more stable than full-length SMAX1 (38), it may be more amenable to chromatin immunoprecipitation analysis and identification of SMAX1 protein partners via coimmunoprecipitation and tandem mass spectrometry. Further refinement of critical features within the N domain that specify developmental roles will also be useful in order to determine how SMXL proteins evolved different functions during the diversification of this family in the angiosperm lineage.

In future studies, it will be important to validate the candidate TF interactions with SMAX1 or SMXL7 through genetic and biochemical approaches, as Y2H analyses can produce false-positive or false-negative results. Of particular interest for further investigation are TFs that may interact differentially with SMAX1 and SMXL7. Our Y2H screen suggested that SMAX1 may interact with multiple members of the TCP, HB, GRAS, bHLH, and AP2/EREBP families (*SI Appendix,* Table S1). TEOSINTE BRANCHED 1/CYCLOIDEA/PCF (TCP) family proteins are categorized into class I-PCF, class II-CIN, and class II-CYC/TB1 subclades. All class I TCPs that we tested, except for TCP21, interacted with both SMAX1 and SMXL7. Most of the tested class II TCPs also interacted with both SMAX1 and SMXL7, but TCP5 and TCP17 only interacted with SMAX1. TCP18/BRC1, a key regulator of branching (74, 80), putatively interacted with SMAX1 and SMXL7 through the N domain. Regarding the GRAS family, we examined four of the five *Arabidopsis* DELLAs. GAI, RGL1, and RGL3 interacted with SMAX1 and SMXL7, but RGA showed SMAX1-specific interaction. The N domain was necessary but not sufficient for SMXL–DELLA interactions, consistent with a prior report that these DELLAs cannot interact with SMAX1 lacking its first 163 amino acids (22). In some cases, our Y2H results differed from expectations. For example, we did not observe clear SMAX1 or SMXL7 interactions with *Arabidopsis* homologs of OsGRF4, OsBZR1, or OsIPA1 (*SI Appendix,* Fig. S10 and Table S1) (21, 23, 25). It is very likely that other interaction partners of SMAX1 or SMXL7 have also been missed due to technical limitations of the Y2H approach and because we did not test all TFs in *Arabidopsis*.

Finally, we showed that chimeric SMXL proteins could be created that crosswire the normal responses to KARs and SLs. For example, the SMXL*χ*177 protein enabled SL-specific control of seed germination and seedling growth (Fig. 2). This suggests that important agronomic traits in crops that are controlled by SMXL proteins, including plant architecture and symbiotic interactions with microbes, could be genetically engineered to be regulated by a different hormone. We also demonstrated the creation of a miniaturized form of SMAX1 through the SMAX1_N210_-SRDX fusion. This protein recapitulates SMAX1 function but escapes SCF^MAX2^-dependent regulation (Fig. 3). It could conceivably be further fused to degrons from plant or nonplant systems to engineer inducible forms of developmental control (81).

## Materials and Methods

### DNA Constructs for Transgenic Plants.

A binary Gateway destination vector, pGWBcitr, was generated by replacing the hygromycin resistance cassette of pGWB501 with a seed coat-specific Citrine cassette from pYUU (82, 83). The promoter upstream of the Gateway cassette was replaced with *Arabidopsis SMAX1* and *SMXL7* promoter (3 kbp of DNA upstream of the translation start site). *Arabidopsis SMAX1* and *SMXL7* coding sequences with C-terminal 3xFLAG tags were cloned into pDONR221 vector by Gateway BP reaction (Invitrogen).

N-terminal SV40 NLS-, C-terminal FLAG-SRDX-fused *SMAX1_N158_*, *SMAX1_N210_*, and *SMXL7_N174_* sequences were synthesized (Twist Biosciences) and cloned into pDONR221 to generate *SMAX1_N158_-SRDX*, *SMAX1_N210_-SRDX*, and *SMXL7_N174_-SRDX*. *SMAX1_N158_, SMAX1_N210_, SMXL7_N174_*, and *SMXL7_N190_* fused with N-terminal NLS and C-terminal FLAG were amplified and introduced into pDONR221 to generate Gateway entry clones without the C-terminal SRDX. *NLS-eGFP-FLAG-SRDX* (GFP-SRDX) and *NLS-eGFP-FLAG* (GFP) were assembled through NEBuilder (New England Biolabs) and cloned by Gateway BP reaction. *SMAX1ΔN* and *SMXL7ΔN* fused with N-terminal SV40 NLS and C-terminal FLAG tag were generated with NEBuilder by replacing *eGFP* from the *GFP* entry clone with N-terminally truncated *SMAX1* (encoding 159 to 990 aa) and *SMXL7* (encoding 175 to 1,002 aa). To generate *SMXLχ177, SMXLχ717, SMXLχ771, SMXLχ711, SMXLχ171,* and *SMXLχ117* fused with C-terminal FLAG tag, the N, D1M, and D2 domains of SMAX1 and SMXL7 were amplified respectively, assembled through overlap-extension PCR, and inserted into the pDONR221 entry vector by BP reaction. Boundaries of the SMAX1 and SMXL7 domains are depicted in *SI Appendix*, Figs. S3 and S4. To generate C-terminal FLAG-fused *SMXL7^ΔRGKT^* and *SMXLχ177^ΔRGKT^*, the RGKT motif was removed through Q5 Site-Directed Mutagenesis Kit (New England Biolabs) from the *SMXL7* and *SMXLχ177* entry clones, respectively. To generate *SMXLχ1_210_77^ΔRGKT^*, the elongated N domain of *SMAX1* (encoding 1 to 210 aa) and the N_190_-truncated SMXL7*^ΔRGKT^* (encoding 191 to 1,002 aa without RGKT motif) fused with C-terminal FLAG were assembled through overlap-extension PCR and cloned into pDONR221 vector.

Inserts in Gateway entry clones were transferred into pGWBcitr-*SMAX1pro* and pGWBcitr-*SMXL7pro* by Gateway LR reaction (Invitrogen). The eYFP-SMXL*χ* fusions used to visualize subcellular localization were made by introducing the series of entry clones containing the intact and chimeric SMXLs into pGWB542 (82) by LR reaction. Primers used for cloning and plasmid construction are listed in *SI Appendix,* Table S3.

### Plant Materials and Growth Conditions.

*A. thaliana* mutants *smax1-2 smxl2-1*, *max3-9*, and *max3-9 smxl6-4 smxl7-3 smxl8-1* (Col-0 ecotype) were described previously (4, 84). *Agrobacterium*-mediated floral dip was performed to transform *smax1,2* or *max3 smxl6,7,8* (85). Homozygous transgenic lines were identified and used in subsequent characterization. Primers used for genotyping are listed in *SI Appendix,* Table S3. Seeds were surface-sterilized, plated on 0.5 × Murashige and Skoog (0.5 × MS) medium with 0.8% (w/v) Bacto agar, and stratified at 4 °C for 3 d before introduction to standard growth conditions (16 h white light, 8 h dark at ~21 C) unless otherwise specified. Plants were grown in Sungro Professional Growing Mix supplemented with Gnatrol WDG (*Bacillus thuringiensis*) and Marathon (imidacloprid).

### Structural Analysis of SMAX1 and SMXL7.

Globular regions of SMAX1 and SMXL7 were predicted with IUPred3 structural domains tool (86). IDRs were predicted using the D^2^P^2^ database (87). Predicted IDRs with over 75% agreement are indicated in *SI Appendix*, Fig. S4*A* and Table S1.

### Chemicals.

GR24^5DS^ was produced by StrigoLab (Torino, Italy). KAR_2_ was synthesized and kindly provided by Dr. Adrian Scaffidi and Dr. Gavin Flematti (University of Western Australia, Crawley, Australia).

### Plant Phenotyping.

Hypocotyl elongation assays were performed with slight modification as previously described (76). Seeds were surface-sterilized, plated on 0.5 × MS media supplemented with 1 µM KAR_2_, GR24^5DS^, or an equivalent volume of acetone solvent, stratified for 3 d at 4 °C in darkness, and moved to a HiPoint DCI-700 LED Z4 growth chamber to grow at 21 °C under white light (150 µmol m^−2^ s^−1^) for 3 h, dark for 21 h, and continuous red light (30 µmol m^−2^ s^−1^) for 6 d. Hypocotyl lengths were measured from photographs of seedlings using ImageJ (NIH). Statistical significance (*P*<0.05) was calculated through Tukey’s multiple comparisons.

For branching and shoot height measurement, seedlings grown on 0.5 × MS for 10 d were moved to soil without fertilizer and grown under 16-h white light/8-h dark cycles at ~21 °C. The primary shoot height and the number of rosette axillary branches at least 10 mm in length of 8-wk-old plants were counted. Statistical significance (*P* < 0.05) was calculated through Tukey’s multiple comparisons.

Germination assays of seeds aged at room temperature for at least one month after harvest were performed as previously described (35). Seeds were surface-sterilized, plated on 0.5 × MS containing 3 µM paclobutrazol (PAC), stratified 4 d at 4 °C in darkness, and then moved to a HiPoint DCI-700 LED Z4 growth chamber to grow at 25 °C under continuous white light (100 µmol m^−2^ s^−1^). After 10 d, germination was scored as radicle emergence.

### Gene Expression Analysis.

Gene expression analyses were performed as previously described with slight modifications (76). For transcriptional analysis of the KAR/KL responsive genes, Col-0, *smax1,2*, transgenic *smax1,2* seedlings expressing *SMAX1pro::SMAX1*, *SMAX1pro::SMXL7*, *SMAX1pro::SMXLχ177,* and *SMAX1pro::SMAX1_N210_-SRDX* were grown as described for hypocotyl elongation assays and harvested after 4 d in red light. The aerial parts of 5-wk-old Col-0 (rosette leaves and whole shoots), *max3 smxl6,7,8*, and transgenic *max3 smxl6,7,8* plants expressing *SMXL7pro::SMAX1*, *SMXL7pro::SMXL7*, *SMXL7pro::SMXLχ711,* and *SMAX1pro::SMXL7* were collected to test transcription of the SL-responsive genes. Total RNA extraction was performed with the Monarch Total RNA Miniprep Kit (New England Biolabs), and cDNA was synthesized using the Verso cDNA Synthesis Kit (Thermo Fisher Scientific). Real-time quantitative PCR was performed on first-strand cDNA with Luna Universal qPCR Mastermix (New England Biolabs) in a CFX384 system (BioRad). A two-step amplification-melt protocol was used with the following conditions: 95 °C for 1 min; 45 cycles of 95 °C for 15 s, 60 °C for 30 s; melt-curve from 65 °C to 95 °C. Primer sequences are described in *SI Appendix*, Table S3. Three to four biological replicates were tested. The average Ct value for each transcript in a biological replicate was determined from two to three technical replicates per reaction. *CACS*/*AP2M* was used as a reference transcript to normalize expression of the tested genes using the 2^-ΔΔCt^ method. Relative expression values were scaled relative to the wild type (Col-0).

### Yeast Two-Hybrid Assays.

*SMAX1, SMAX1ΔN, SMAX1_N158_, SMAX1_N210_, SMXL7, SMXL7ΔN, SMXL7_N174_,* and *SMXL7_N190_* in Gateway entry clones were transferred into pDEST32 (Invitrogen) by LR reaction. Bait plasmids were introduced into the Y2HGold yeast strain (Takara) using the lithium acetate method (88). Prey plasmids from the pDEST22-*Arabidopsis* TF library (78) were introduced into bait-transformed yeast lines. Cotransformed yeast were selected through growth on −Leu/−Trp (−LW) synthetic dropout media for 3 d at 30 °C. Bait–prey interactions were examined by spotting cells (10 μL of colony suspension at OD_600_ 0.15) on −LW and −Leu/−Trp/−His (−LWH) synthetic dropout plates. After 3 d at 30 °C, plates were photographed and colony growth was quantified using a modified density analysis method (89). Briefly, photographs were converted to grayscale, and gray values of yeast spots and their backgrounds were measured using ImageJ (NIH). Background values were subtracted from yeast spot values on the same plate. The background-subtracted values from −LWH plates were divided by the corresponding values from −LW plates. The ratios for the two colony replicates in each test were log_2_ transformed and averaged. Values above -0.5 were considered a positive interaction.

### Subcellular Localization Analysis.

*A. tumefaciens* strain GV3101 carrying pGWB542 expression clones with *SMAX1*, *SMXL7*, and *SMXLχ* variants were infiltrated into *N. benthamiana* as previously described (90). The eYFP, 4′,6-diamino-2-phenylindole dihydrochloride (DAPI) and propidium iodide (PI) fluorescent signals were visualized using 880 Inverted Airyscan Fast confocal microscope (Zeiss) with the setting of eYFP (excitation, 514 nm; emission, 527 nm), DAPI (excitation, 405 nm; emission, 488 nm), and PI (excitation, 535/20 nm; emission, 610/20). For costaining, leaf discs were incubated in distilled water supplemented with 10 µg/mL DAPI and 10 µg/mL PI for 20 min in darkness.

### Gene Accession Numbers.

SMAX1 (AT5G57710.1), SMXL2 (AT4G30350.1), SMXL6 (AT1G07200.2), SMXL7 (AT2G29970.1), SMXL8 (AT2G40130.2), OsD53 (Os11g01330.1), OsSMAX1L (Os08g15230.1), OsSMXL2 (Os02g54720.1), DLK2 (AT3G24420), KUF1 (AT1G31350), ERF61 (AT4G17490), BBX20/STH7 (AT4G39070), IAA29 (AT4G32280), BRC1/TCP18 (AT3G18550), PAP1 (AT1G56650), TCP1 (AT1G67260), and CACS/AP2M (AT5G46630). Accession numbers for TFs used in the Y2H assay are listed in *SI Appendix,* Table S2.

## Supplementary Material

Appendix 01 (PDF)

## Data Availability

All study data are included in the article and/or *SI Appendix*.
