# Supplementary material for: An N-terminal domain specifies developmental control by the SMAX1-LIKE family of transcriptional regulators in Arabidopsis thaliana
Source: Proc Natl Acad Sci U S A. 2025 Jun 10;122(24):e2412793122. doi: 10.1073/pnas.2412793122 (PMC12184505; doi:10.1073/pnas.2412793122)
Supplement: Supplementary file 1 — Appendix 01 (PDF) [file pnas.2412793122.sapp.pdf]

PNAS

Supporting Information for
An N-terminal domain specifies developmental control by the SMAX1-LIKE family of transcriptional regulators in *Arabidopsis thaliana*

Sun Hyun Chang[1], Wesley George[1], David C. Nelson[1]*

[1]Department of Botany and Plant Sciences, University of California, Riverside, 92521 USA

*David C. Nelson

Email: david.nelson@ucr.edu

This PDF file includes:

Figures S1 to S11
Tables S1 to S3
SI References

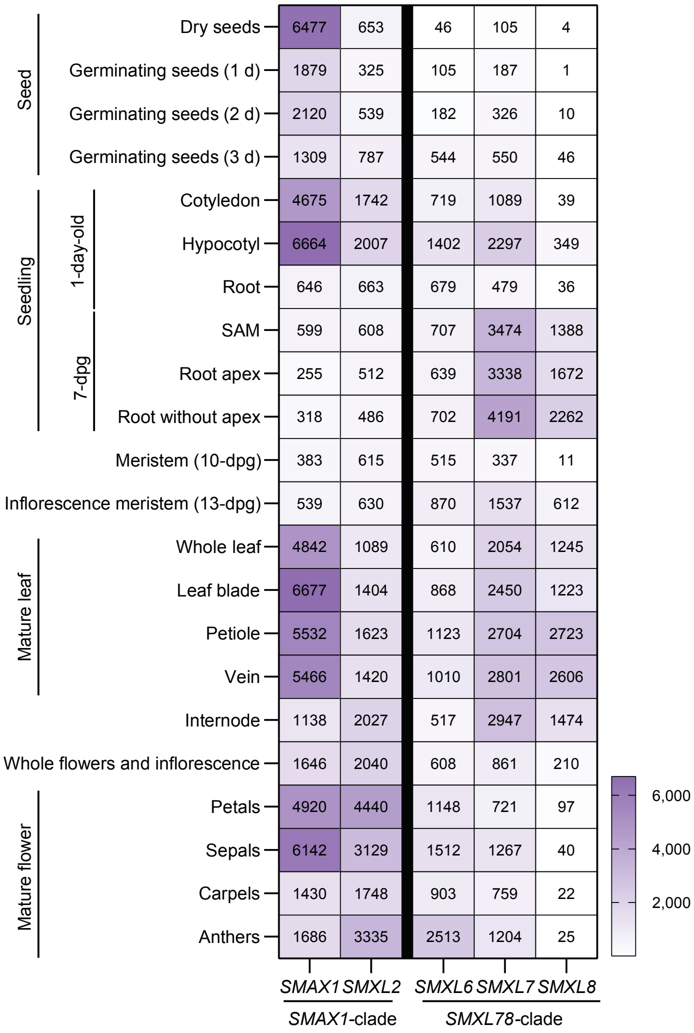

Fig. S1. Expression pattern of *SMAX1*- and *SMXL78*-clade genes at different stages of *Arabidopsis* development.

Overview of mRNA expression of *SMAX1* and *SMXL78* clades using TraVA (travadb.org). The number in each box represents the normalized average count per million reads for each gene in the corresponding sample. 'Seed' samples were collected on the specified days after germination. Seedling samples labeled '1-day-old' were collected from one-day-old seedlings, and '7-dpg' samples were collected from the whole or specified tissues of the third leaf at the time of anthesis of the first flower. All the samples categorized in 'Mature leaf' were collected from the whole or specified tissue of the third leaf at the time of anthesis of the first flower. The 'internode' sample represents the first elongated internode between the last rosette leaf and the first cauline leaf, collected at the time of the anthesis of the first flower. 'Whole flowers and inflorescence' represents the average expression of flowers collected at the time of anthesis of the first flower. Samples of 'Mature flower' were collected from the specified floral parts collected at the moment of the anthesis of the first flower. 'D' means day, and 'dpg' means day post-germination. More detailed information on the samples can be found in http://travadb.org/samples/.

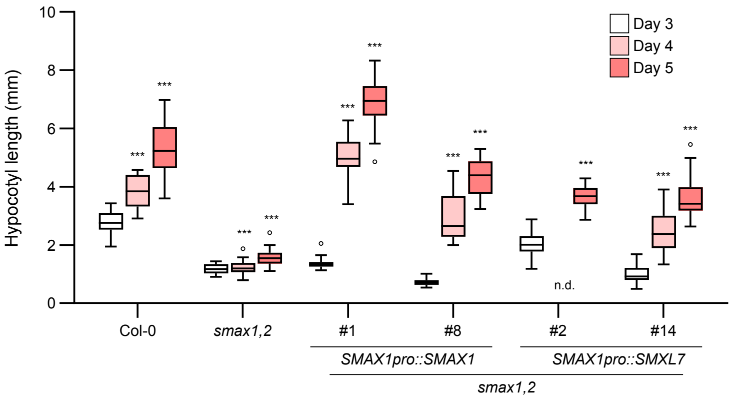

Fig. S2. Hypocotyl elongation dynamics in *SMAX1*- and *SMXL7*-expressing *smax1,2* transgenic seedlings

Hypocotyl lengths in Col-0, *smax1,2,* transgenic seedlings expressing *SMAX1pro::SMAX1* (lines #1 and #8) and *SMAX1pro::SMXL7* (lines #2 and #14) in the *smax1,2* background were measured. Seedlings were stratified in the dark for 3 d at 4 C, then moved to 21 C and treated with 3 h white light, 21 h dark, followed by continuous red light. Hypocotyl lengths were measured after three to five days of growth under red light (n=20 seedlings). Boxplots show mean with quartiles and Tukey's whiskers; open symbols are outlier points that fall beyond the range of the whiskers. Asterisks indicate significant differences compared to day 3 within each line (P<0.0001, two-way ANOVA followed by Tukey's multiple comparisons test). n.d., no data.

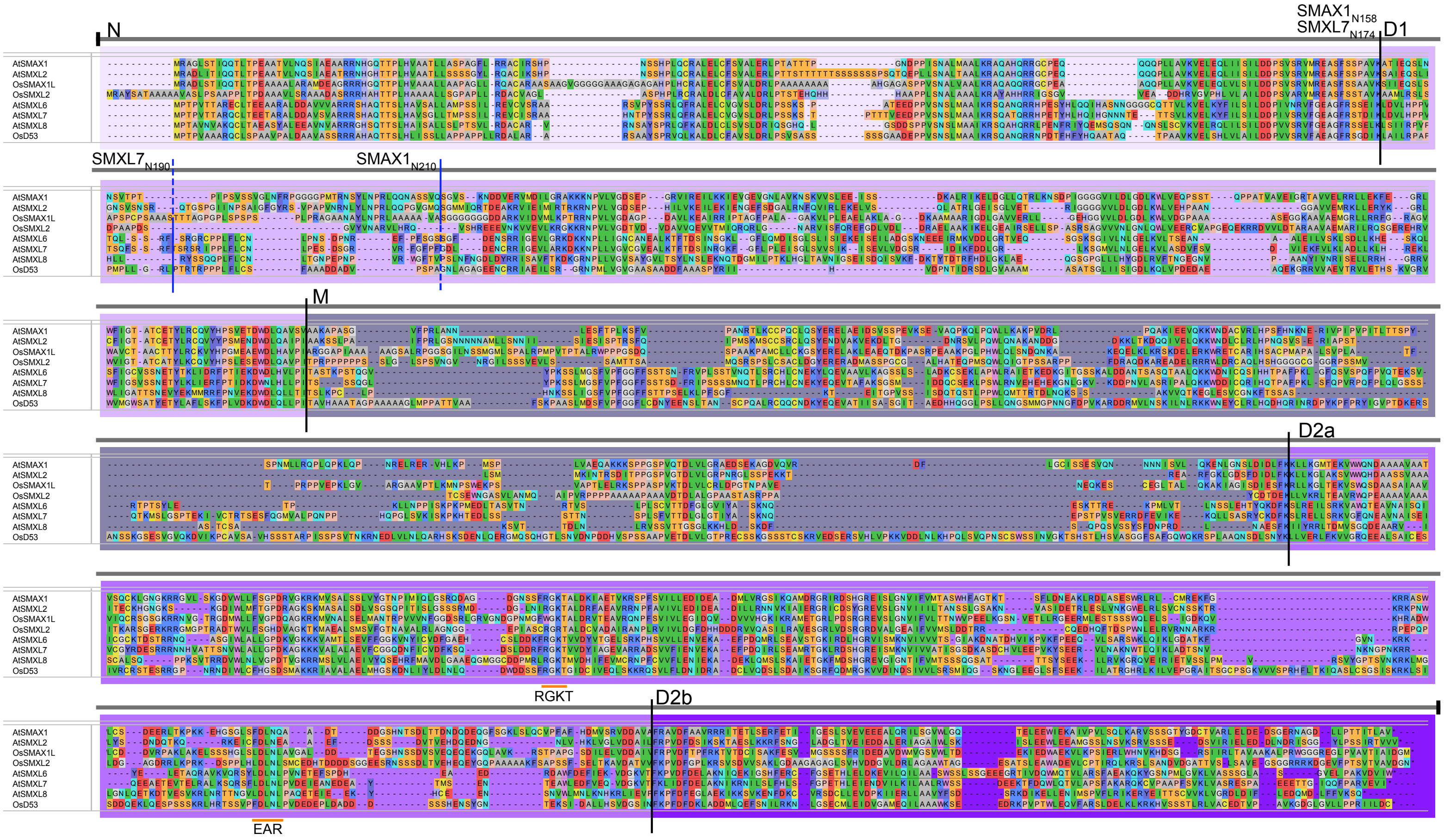

Fig. S3. Domain boundaries of SMAX1- and SMXL78-clade proteins in *Arabidopsis* and rice.
Multiple sequence alignment of SMAX1 and SMXL78-clade protein sequences from *Arabidopsis thaliana* and *Oryza sativa* (rice) to show the domain boundaries. The start positions of the N, D1, M, D2a, and D2b domains are indicated by black vertical bars and each domain is color-labeled with different purples. The extended boundaries for $SMAX1_{N210}$ and $SMXL7_{N190}$ are marked with blue bars. RGKT motif and EAR motif are orange highlighted. The residues are highlighted based on their chemical properties. The alignment was performed using Clustal Omega in DNASTAR MegAlign Pro software.

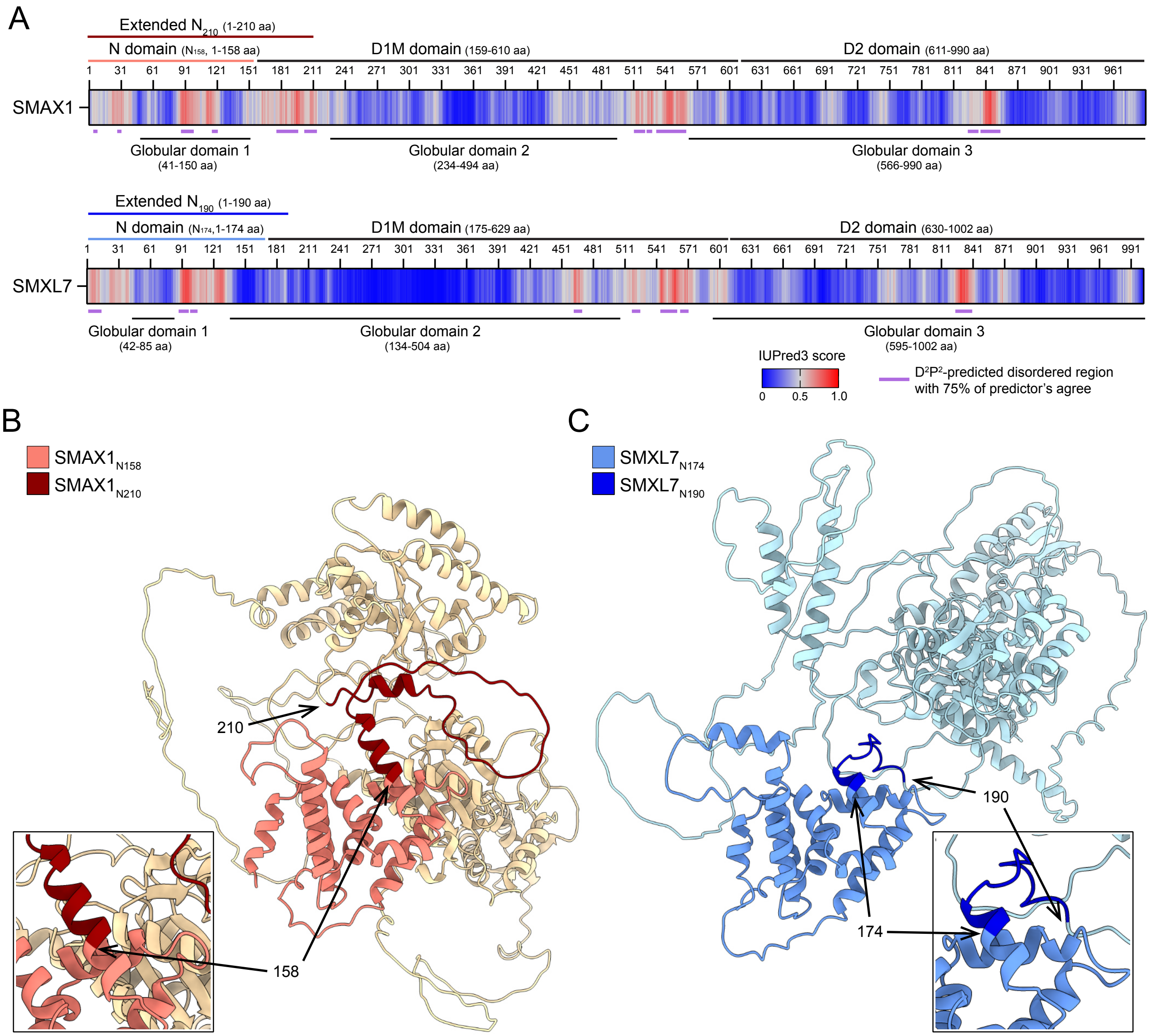

Fig. S4. The boundaries of the defined N domains for SMAX1 and SMXL7 do not include the entirety of the last predicted alpha helix in the domain.

(*A*) The prediction of globular domains and IDRs of SMAX1 and SMXL7. Globular domains were predicted using IUPred3, and disordered regions were predicted using D^2P^2. D^2P^2-predicted IDRs longer than 5 amino acids are represented by purple bars in the figure. Their detailed positions are listed in *SI Appendix, Table S1*. (*B-C*) The Alphafold2-predicted structures of SMAX1 (*B*) and SMXL7 (*C*). The N domains of SMAX1 ($SMAX1_{N158}$, salmon pink) and SMXL7 ($SMXL7_{N174}$, light blue) end at position 158 aa and 174 aa, respectively. The extended N domains ($SMAX1_{N210}$ in dark red and $SMXL7_{N190}$ in dark blue) terminate at positions 210 aa and 190 aa, respectively. Inset boxes show magnified views of the alpha helix regions at these domain boundaries.

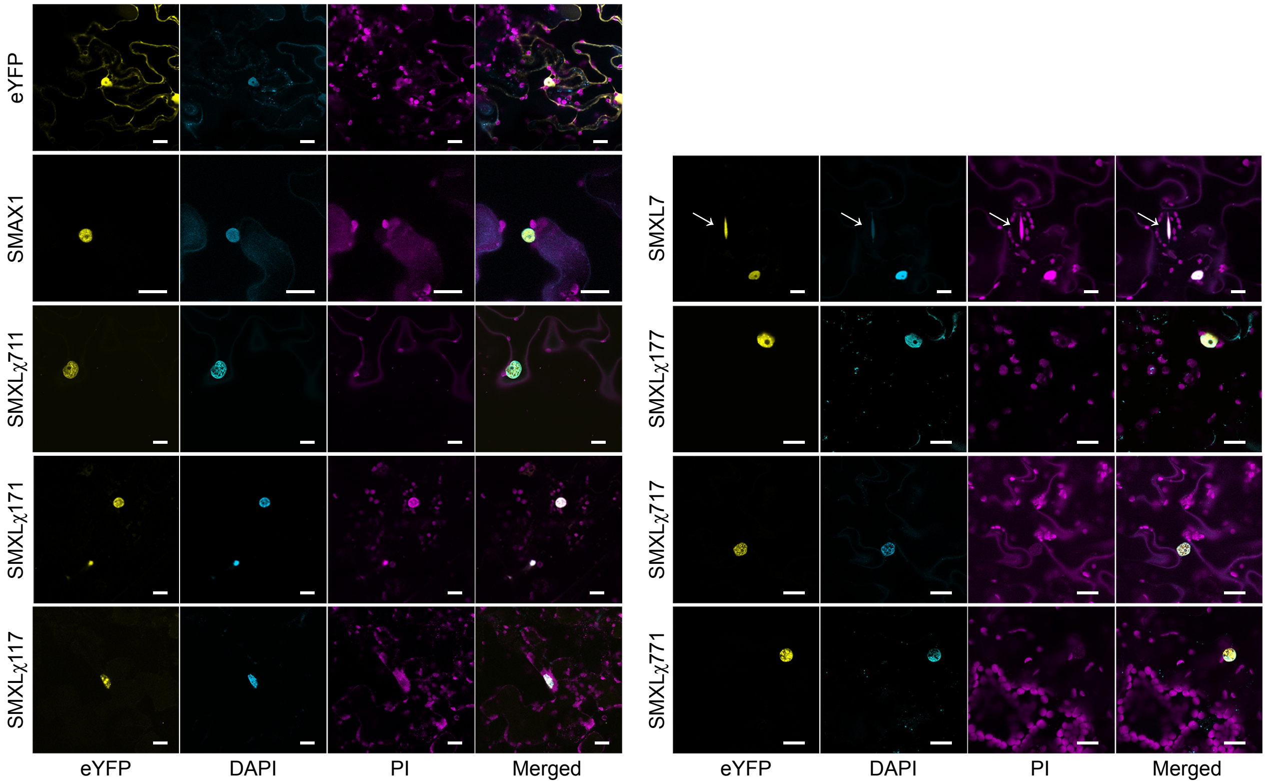

Fig. S5. Subcellular localization of eYFP-tagged chimeric SMXLs in *N. benthamiana*
Confocal microscopy images of transiently expressed N-terminal eYFP-tagged SMAX1, SMXL7, and chimeric SMXLs in *N. benthamiana*. The leaf discs were stained with DAPI and PI. Arrows indicate stomata. Scale bar = 20 μm.

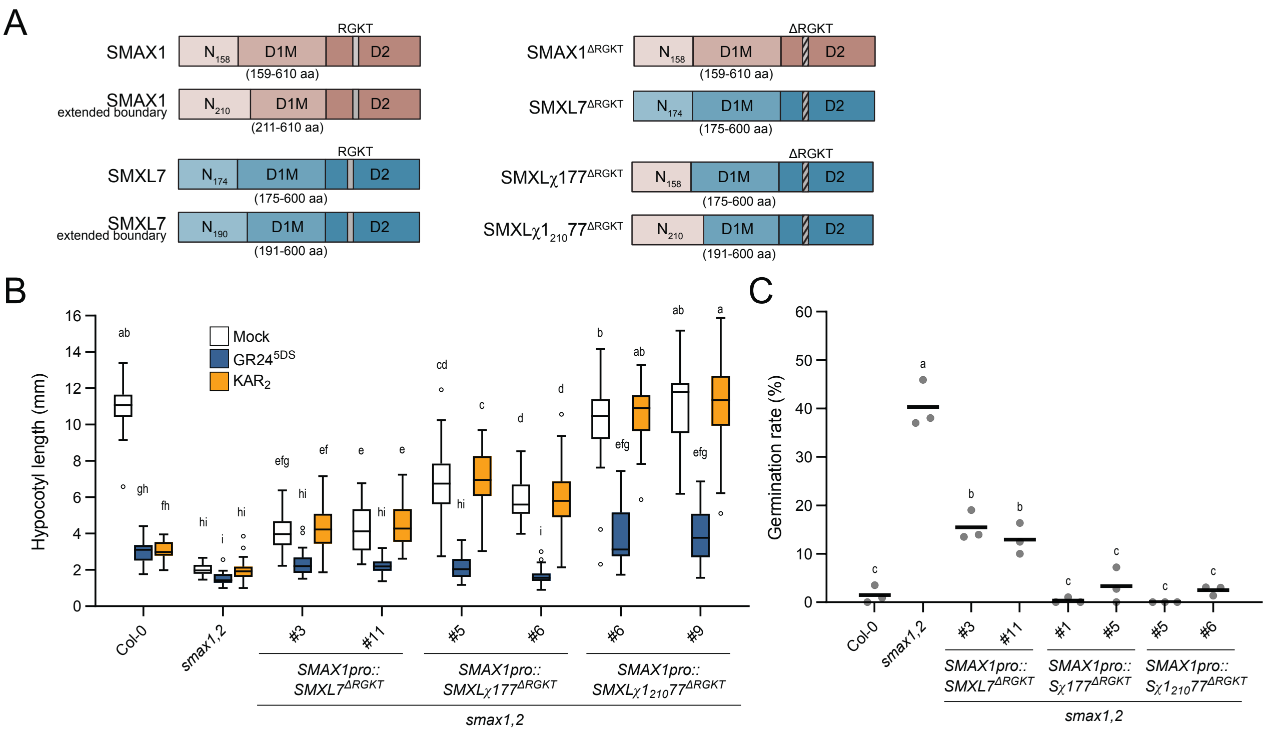

Fig. S6. Seed and seedling growth control by SMXL N domains
(*A*) Diagram of SMAX1, SMXL7, SMXLχ177, SMXL$\chi1_{210}77$, and their RGKT motif-deleted versions $SMAX1^{\Delta RGKT}$, $SMXL7^{\Delta RGKT}$, $SMXL\chi177^{\Delta RGKT}$, and $SMXL\chi1_{210}77^{\Delta RGKT}$. The positions of the RGKT motif and the D1M domain boundaries are indicated. (*B*) Hypocotyl length of Col-0, *smax1,2*, and transgenic lines expressing $SMXL7^{\Delta RGKT}$, $SMXL\chi177^{\Delta RGKT}$, and $SMXL\chi1_{210}77^{\Delta RGKT}$ under control of the *SMAX1* promoter with mock, 1 µM KAR_2, or 1 µM $GR24^{5DS}$ treatment (n≥20). (*C*) Germination of the lines in *B* (n=3, ≥50 seeds per replicate). Boxplots indicate mean with quartiles and Tukey's whiskers; open symbols are outlier points that fall beyond the range of the whiskers. Letters indicate groups with significant differences (P<0.05, two-way ANOVA in (*B*), or one-way ANOVA in (*C*), followed by Tukey's multiple comparisons test).

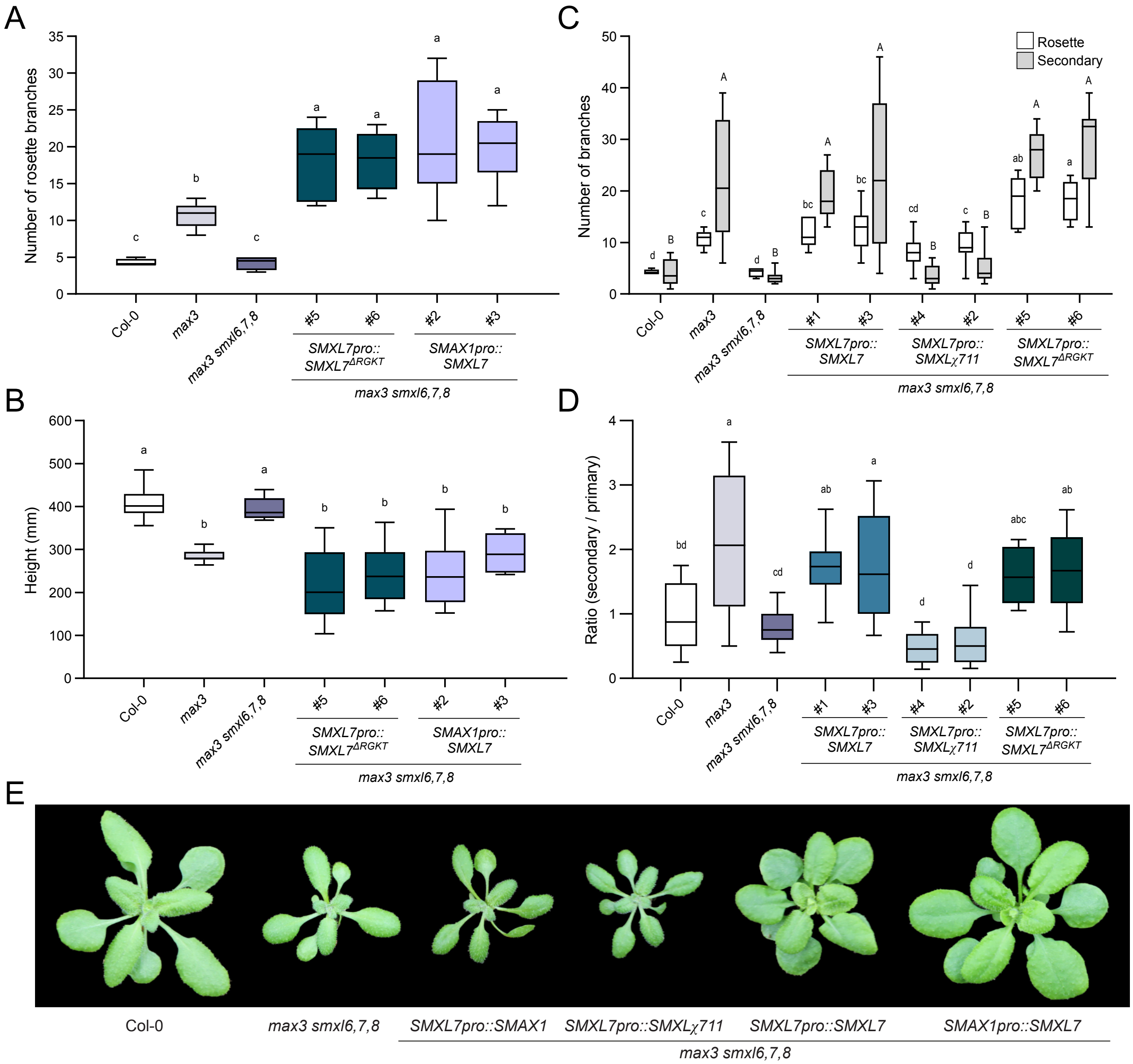

Fig. S7. $SMXL7_N$ is not sufficient to rescue *max3 smxl6,7,8*

(*A*) Number of axillary branches in transgenic lines expressing *SMXL7pro::SMXL7*$^{\Delta RGKT}$ and *SMAX1pro::SMXL7 in max3 smxl6,7,8* (n≥9). Data for the Col-0, *max3*, and *max3 smxl6,7,8* control plants is duplicated in Fig. 2. (*B*) Plant height of the lines used in *A*. (*C*) Number of rosette branches (white) and secondary branches (gray) of the indicated transgenics. Data for the *SMXL7pro::SMXL7* and *SMXL7pro::SMXLχ711* lines is duplicated in Fig. 2*E*, and data for the *SMXL7pro::SMXL7*$^{\Delta RGKT}$ lines is duplicated in *A-B*. (*D*) Ratio of secondary branches to rosette branches in the lines used in *C*. (*E*) Representative image of 3-week-old rosette of Col-0, *smax1,2*, and transgenic lines expressing *SMXL7pro::SMAX1*, *SMXL7pro::SMXLχ711*, *SMXL7pro::SMXL7*, and *SMAX1pro::SMXL7* in *max3 smxl6,7,8* background. Boxplots indicate mean with quartiles and Tukey's whiskers; open symbols are outlier points that fall beyond the range of the whiskers. Letters indicate groups with significant differences ($P<0.05$, one-way ANOVA followed by Tukey's multiple comparisons test). In (*C*), significant differences were calculated separately for rosette branches (uppercase letters) and secondary branches (lowercase letters).

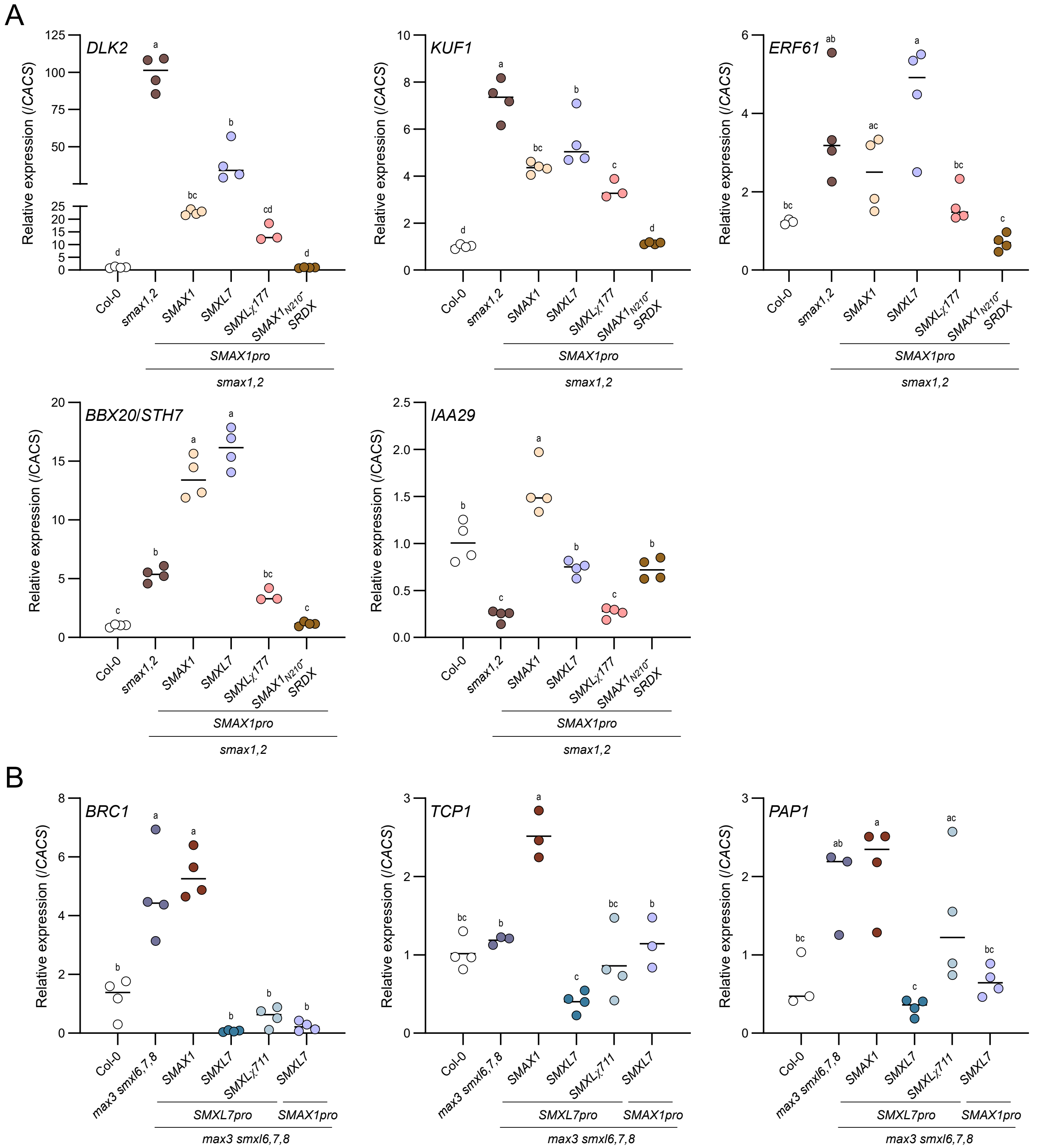

Fig. S8. Transcriptional regulation mediated by the N domain of SMAX1 and SMXL7

(*A*) The expression of KAR/KL-responsive marker genes *DLK2*, *KUF1*, *ERF61*, *BBX20*/*STH7*, and *IAA29* were analyzed in Col-0, *smax1,2*, and transgenic *smax1,2* seedlings expressing *SMAX1pro::SMAX1*, *SMAX1pro::SMXL7*, *SMAX1pro::SMXLχ177, and SMAX1pro::SMAX1*$_{N210}$*-SRDX* grown in red light through qRT-PCR. (*B*) The expression of SL marker gene *BRC1*, *TCP1*, and *PAP1* was analyzed in the aerial parts of 5-week-old Col-0, *max3 smxl6,7,8,* and transgenic lines expressing *SMXL7pro::SMAX1*, *SMXL7pro::SMXL7, SMXL7pro::SMXLχ711*, and *SMAX1pro::SMXL7* in *max3 smxl6,7,8* background. Expression of the target genes were normalized with *CACS*/*AP2M.* Each dot indicates independent biological replicate (n=3-4). Letters indicate groups with significant differences (P<0.05, one-way ANOVA followed by Tukey's multiple comparisons test).

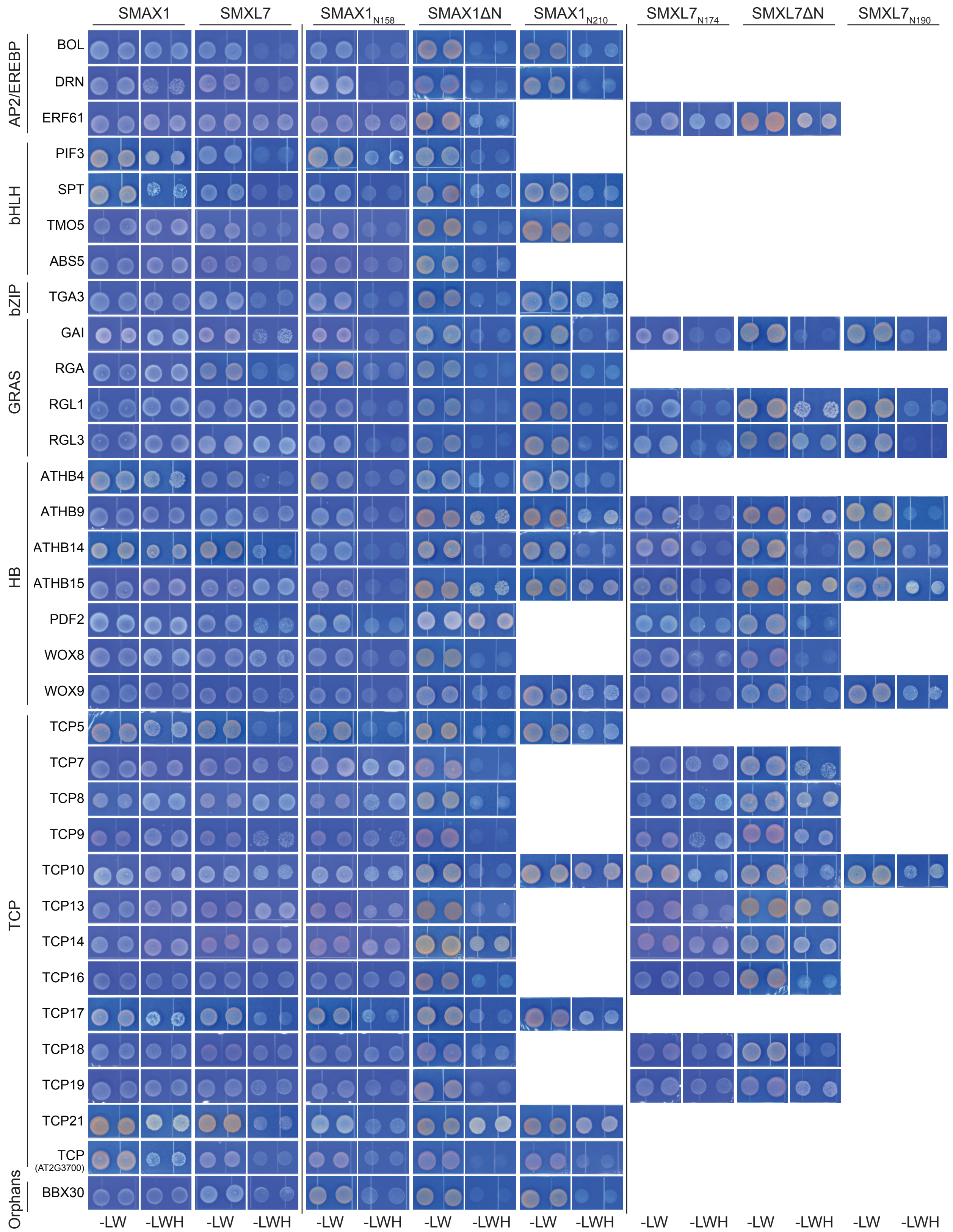

Fig. S9. Photograph of Y2H results with TFs showing potential interactions with SMAX1 and/or SMXL7. Interactions between SMAX1, SMXL7 and their truncated variants fused to GAL4-BD and candidate TFs fused to GAL4-AD were tested. Two replicates were spotted onto selective growth medium (-L, -Leu; -W, -Trp; -H, -His), incubated 3 d at 30℃, and photographed.

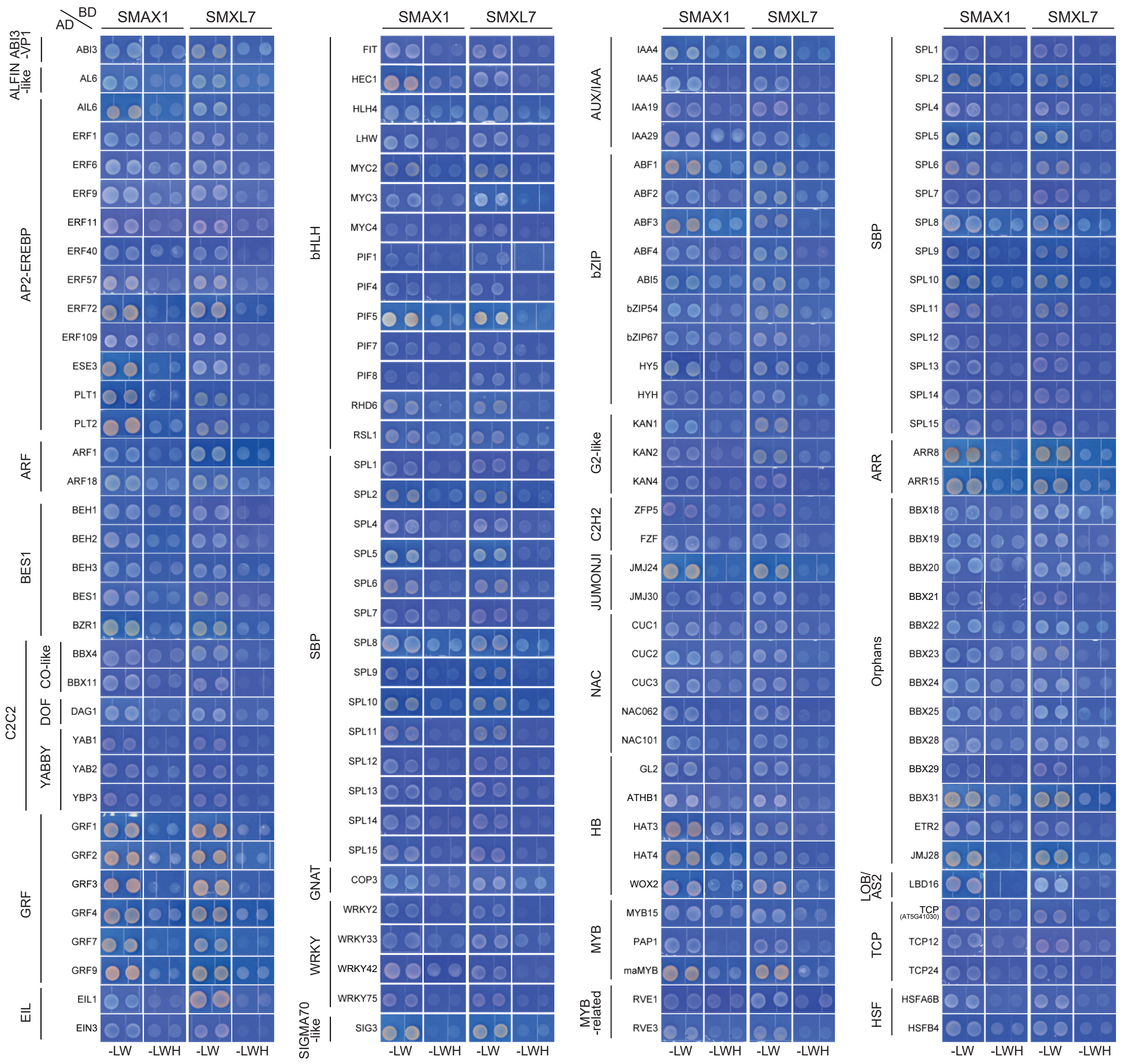

Fig. S10. Y2H assays that showed no interaction between SMAX1 and candidate TFs.
Y2H assay results showing no interaction between SMAX1 or SMXL7 and the indicated TFs fused to GAL4-AD.

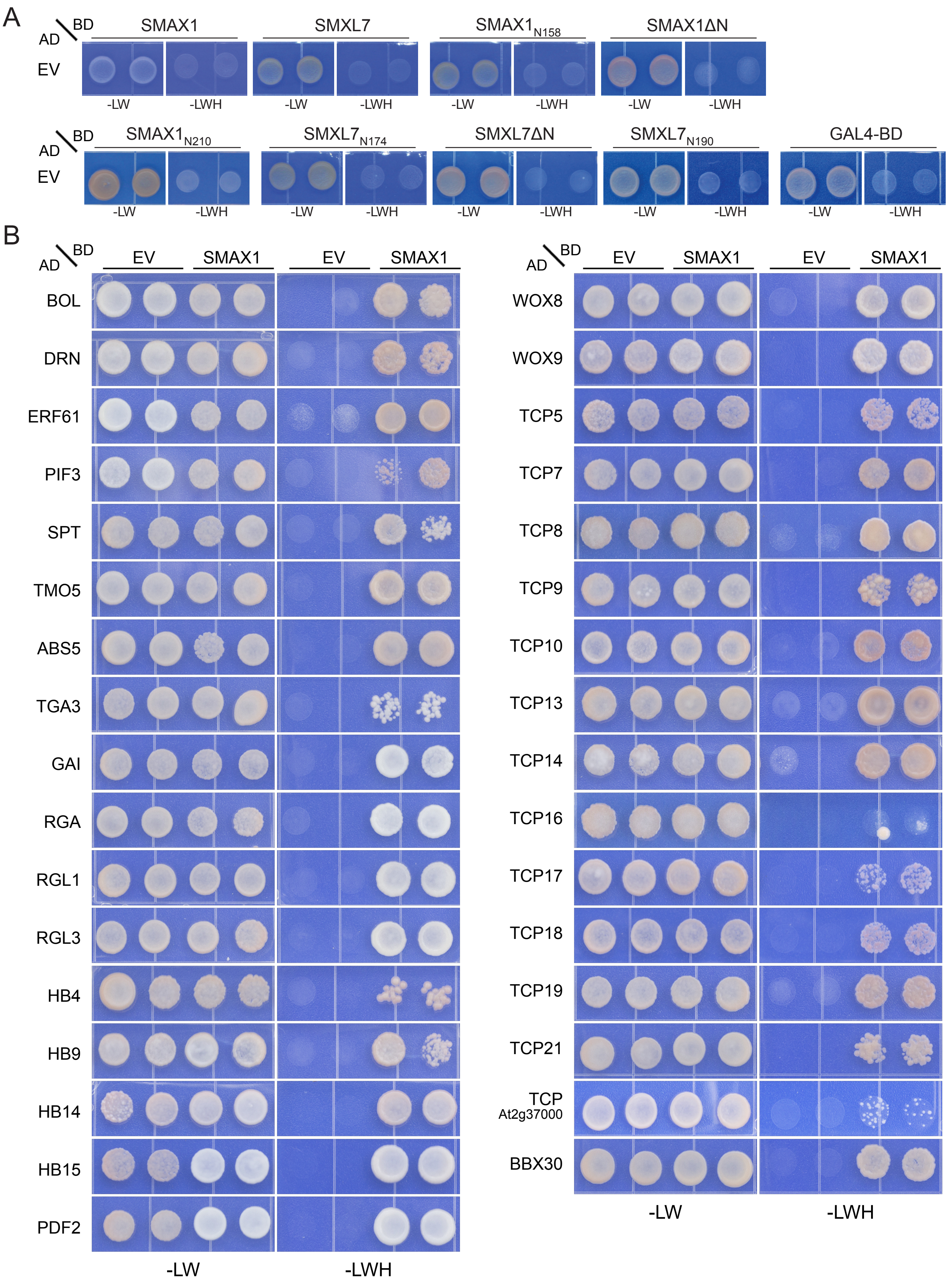

Fig. S11. Self-activation tests of TF preys that interact with SMAX1
(*A*) Autoactivation test for SMAX1, SMXL7, and their truncated versions fused with the GAL4-BD. EV indicates empty prey plasmid pDEST22. (*B*) Autoactivation test for prey TFs that showed interactions with SMAX1. EV indicates empty bait plasmid pDEST32.

Table S1. Details of disorder prediction for SMAX1 and SMXL7 by D^2P^2

**Protein**	ATSMAX1
**Accession**	AT5G57710.1
**Protein sequence***	MRA**GLSTI**QQT**L**TPEAATVLNQSIA**EAARRN**HGQTTPLHVAATLLASPAGFLRRACIRSHPNSSHPLQCR<br>ALELCFSVALERLPT**ATTTPGNDPPISN**ALMAALKRAQAHQRRGCP**EQQQQ**PLLAVKVELEQLIISILDDPS<br>VSRVMREASFSSPAVKAT**IEQSLNNSVT**P**T**PIP**SVSSVGLNFRP**G**GGGPMTR**NSYLNPR**LQQNASSVQS**<br>**GVS**KNDDVERVMDILGRAKKKNPVLVGDSEPGRVIREILKKIEVGEVGNLAVKNSKVVSLEEISSDKALRIK<br>ELDGLLQTRLKNSDPIGGGGVILDLGDLKWLVEQPSSTQPPATVAVEIGRTAVVELRRLLEKFEGRLWFIG<br>TATCETYLRCQVYHPSVETDWDLQAVSVAAKAPASGVFPRLANNLESFTPLKSFVPANRTLKCCPQCLQ<br>SYERELAEIDSVSSPEV**K**SEVAQPKQLPQWLLKAKPVDRLPQAKIEEVQKKWNDACVRLHPSFHNKNERI<br>VPIPVPITLTTSPYS**P**N**MLLRQPLQPKL**Q**PNRE**LRERVH**LKPMSPLVAEQAKKKSPPGSPVQTDLVLGR**<br>AE**D**SEKAGDVQVRDFLGCISSESVQNNNNISVLQKENLGNSLDIDLFKKLLKGMTEKVWWQNDAAAAVA<br>ATVSQCKLGNGKRRGVLSKGDVWLLFSGPDRVGKRKMVSALSSLVYGTNPIMIQLGSRQDAGDGNSSF<br>RGKTALDKIAETVKRSPFSVILLEDIDEADMLVRGSIKQAMDRGRIRDSHGREISLGNVIFVMTASWHFAGT<br>KTSFLDNEAKLRDLASESWRLRLCMREKFGKRRASWLCSDEERLTKP**KKEH**GSG**LSFDLNQAA**DT**DDG**<br>**SHNTSDLTTDNDQD**EQGFSGKLSLQCVPFAFHDMVSRVDDAVAFRAVDFAAVRRRITETLSERFETIIGE<br>SLSVEVEEEALQRILSGVWLGQTELEEWIEKAIVPVLSQLKARVSSSGTYGDCTVARLELDEDSGERNAG<br>DLLPTTITLAV

* The **highlighted portions of sequence** are where there is 75% agreementa between all predictors in the database for this region being disordered.

Predicted Disordered Regions**

Start	End
4	8
12	12
26	31
86	98
117	121
161	170
172	172
176	186
188	194
202	214
441	441
509	509
511	521
523	526
533	562
565	565
819	822
826	834
837	853

* Regions over 75% agreement between all predictors in the database for this region being disordered.

List of Disordered Regions by Predictor:

Predictor	Start	End
VLXT	1	2
VSL2b	1	20
PrDOS	1	13
PV2	1	39
IUPred-S	1	9
Espritz-N	1	13
Espritz-X	1	8
IUPred-L	3	7
VLXT	4	31
IUPred-L	12	12
IUPred-L	19	33
VSL2b	23	39
PrDOS	25	32
Espritz-N	26	37
IUPred-S	28	28

IUPred-S	30	31
IUPred-L	35	39
VLXT	36	36
PV2	48	48
VSL2b	56	68
PV2	56	61
PrDOS	58	68
Espritz-N	58	66
PV2	63	68
PrDOS	81	123
Espritz-N	81	98
VSL2b	82	129
PV2	82	131
VLXT	84	104
IUPred-L	84	84
IUPred-S	86	105
IUPred-L	86	103
Espritz-X	88	90
Espritz-N	105	122
IUPred-S	107	116
IUPred-L	107	121
VLXT	117	126
IUPred-S	118	118
VLXT	133	139
VSL2b	134	135
PV2	134	135
VSL2b	138	245
PV2	138	237
Espritz-N	141	142
IUPred-L	148	149
VLXT	149	254
Espritz-N	150	194
IUPred-L	157	157
PrDOS	160	219
IUPred-L	160	170
IUPred-S	170	170
IUPred-S	172	172
IUPred-L	172	172
IUPred-L	175	186
IUPred-S	180	216
IUPred-L	188	214
Espritz-N	202	218
IUPred-L	223	224
IUPred-S	225	225
Espritz-N	229	242
IUPred-L	233	233
IUPred-L	237	237
PV2	240	241
PV2	244	244
VSL2b	254	254
PrDOS	255	266
VSL2b	256	260
VSL2b	262	280
PrDOS	272	283
VLXT	273	292
PV2	277	280
Espritz-N	294	303

VSL2b	295	297
PV2	301	304
PV2	315	329
VSL2b	318	325
Espritz-N	319	321
PrDOS	320	334
VLXT	321	339
IUPred-S	326	326
PrDOS	383	414
PV2	387	389
VSL2b	413	413
VSL2b	415	415
VSL2b	417	471
PV2	418	472
VLXT	424	442
PrDOS	431	470
Espritz-N	435	450
IUPred-L	440	441
IUPred-S	443	443
VLXT	452	471
IUPred-L	466	467
PV2	474	476
PrDOS	482	606
Espritz-N	484	526
PV2	490	490
VLXT	497	567
IUPred-L	498	500
PV2	499	575
VSL2b	502	575
IUPred-L	505	505
IUPred-L	507	509
IUPred-S	511	511
IUPred-L	511	521
IUPred-S	513	563
IUPred-L	523	562
Espritz-N	533	557
Espritz-X	541	561
Espritz-N	560	570
IUPred-S	565	565
IUPred-L	565	565
VLXT	573	573
PV2	582	592
VSL2b	584	592
Espritz-N	586	593
IUPred-L	592	592
IUPred-L	594	594
VSL2b	596	598
PV2	596	598
Espritz-N	597	602
PrDOS	637	652
VLXT	638	649
VSL2b	642	643
VLXT	656	656
VSL2b	665	666
VLXT	669	688
PV2	680	681
PV2	683	687

PrDOS	687	705
IUPred-S	688	689
Espritz-N	688	701
VSL2b	690	702
PV2	690	702
Espritz-X	693	695
IUPred-S	695	695
IUPred-S	697	698
VLXT	700	706
PV2	707	709
VLXT	715	722
VLXT	729	747
PV2	730	730
VSL2b	739	750
IUPred-L	743	743
PV2	744	746
Espritz-N	746	750
PrDOS	767	872
VSL2b	779	779
PV2	784	784
Espritz-N	784	788
VLXT	785	820
VSL2b	792	859
PV2	792	794
PV2	797	860
Espritz-N	798	865
Espritz-X	801	864
IUPred-S	820	855
IUPred-L	820	822
IUPred-L	826	834
IUPred-L	837	853
VLXT	840	854
VLXT	901	927
VSL2b	913	917
PV2	916	916
VSL2b	952	956
PV2	953	953
PrDOS	954	962
Espritz-N	954	959
VLXT	962	981
PV2	966	982
PrDOS	968	990
VSL2b	969	979
Espritz-N	973	978
Espritz-X	975	981
IUPred-S	984	990
Espritz-X	987	990
VSL2b	988	990
PV2	988	990

**Protein**	ATSMXL7
**Accession**	AT2G29970.1
**Protein sequence***	**MPTPVTT**ARQCLTEETARALDDAVSVARRRSHAQTTSLHAVSGLLTMPSSILREVCISRAAHNTPYSSRLQ<br>FRALELCVGVSLDRLP**SSKSTPTTTVEEDPPVS**NSLMAA**IKRSQ**ATQRRHPETYHLHQIHGNNNTETTSVL<br>KVELKYFILSILDDPIVSRVFGEAGFRSTDIKLDVLHPPVTSQFSSRFTSRSRIPPLFLCNLPESDSGRVRFG<br>FPFGDLDENCRRIGEVLARKDKKNPLLVGVCGVEALKTFTDSINRGKFGFLPLEISGLSVVSIKISEVLVDG<br>SRIDIKFDDLGRLKSGMVLNLGELKVLASDVFSVDVIEKFVLKLADLLKLHREKLWFIGSVSSNETYLKLIER<br>FPTIDKDWNLHLLPITSSSQGLYPKSSLMGSFVPFGGFFSSTSDFRIPSSSSMNQTLPRCHLCNEKYEQEV<br>TAFAKSGSMIDDQCSEKLPSWLRNVEHEHEKG**NLGKVKDD**PNVLASRIPALQKKWDDICQRIHQTPAFPK<br>LSFQPVRPQFPLQLG**SSSQTK**MSL**GS**PTEKIVCTRTSESFQG**MVALPQNPPHQPGLSV**KISK**PKHTEDLS**<br>SSTTNSPLSFVTTDLGLGTIYASKN**QEPS**TP**VSVE**RRDFEVIKEKQLLSASRYCKDFKSLRELLSRKVGFQ<br>NEAVNAISEIVCGYRDESRRRNNHVATTSNVWLALLGPDKAGKKKVALALAEVFCGGQDNFICVDFKSQD<br>SLDDRFRGKTVVDYIAGEVARRADSVVFIENVEKAEFPDQIRLSEAMRTGKLRDSHGREISMKNVIVVATIS<br>GSDKASDCHVLEEPVKYSEERVLNAKNWTLQIKLADTS**NVNKNGPNKRRQEEAETE**VTELRALKSQRSF<br>LDLNLPVDEIEAN**EDE**AYTMSENTEAWLEDFVEQVDGKVTFKLIDFDELAKNIKRNILSLFHLSFGPETHLEI<br>ENDVILKILAALRWSSDEEKTFDQWLQTVLAPSFAKARQKCVPAAPFSVKLVASRESPAEE**ETT**GIQQFPA<br>RVEVI

* The **highlighted portions of sequence** are where there is 75% agreement between all predictors in the database for this region being disordered.

Predicted Disordered Regions**

Start	End
1	7
88	104
111	115
464	471
517	522
526	527
544	559
564	571
597	600
603	606
823	840
867	869
988	990

** Regions over 75% agreement between all predictors in the database for this region being disordered.

List of Disordered Regions by Predictor:

Predictor	Start	End
VLXT	1	9
VSL2b	1	43
PrDOS	1	11
PV2	1	43
IUPred-S	1	10
IUPred-L	1	14
Espritz-N	1	6
Espritz-X	1	7
IUPred-S	12	12
IUPred-L	22	33
Espritz-N	32	34
IUPred-L	35	40
VLXT	36	45
PrDOS	54	69
Espritz-N	61	66
PV2	64	68
PV2	71	72
PV2	75	78
PV2	80	138
Espritz-N	82	104
VSL2b	83	126
VLXT	86	117
PrDOS	86	143

Espritz-X	87	97
IUPred-L	88	115
IUPred-S	89	131
Espritz-N	111	137
IUPred-L	118	132
VSL2b	131	138
PrDOS	164	222
PV2	174	177
VSL2b	181	215
PV2	181	207
Espritz-N	191	195
Espritz-N	207	209
PV2	209	211
VSL2b	217	217
PV2	222	222
VLXT	227	237
PrDOS	284	292
PV2	317	317
PV2	324	327
Espritz-N	377	388
VLXT	378	383
VSL2b	378	419
PrDOS	378	416
PV2	380	381
PV2	383	384
PV2	389	391
PV2	393	399
Espritz-N	396	417
PV2	401	406
PV2	409	417
PV2	419	419
VSL2b	427	432
PV2	427	432
PrDOS	431	482
Espritz-N	436	444
PV2	438	438
VSL2b	440	445
PV2	440	477
VLXT	450	453
IUPred-L	452	453
VSL2b	455	471
IUPred-L	456	456
Espritz-N	458	473
IUPred-L	460	474
IUPred-S	463	465
VLXT	464	479
IUPred-S	467	469
PV2	480	480
PV2	483	484
Espritz-N	485	485
PrDOS	494	585
Espritz-N	494	532
VSL2b	496	608
PV2	496	606
IUPred-S	501	501
IUPred-L	501	501
IUPred-L	510	511

IUPred-S	513	516
IUPred-L	513	513
IUPred-L	515	522
VLXT	517	527
IUPred-S	518	524
IUPred-L	524	529
IUPred-S	526	531
IUPred-S	534	537
Espritz-N	535	581
IUPred-S	543	576
IUPred-L	543	576
VLXT	544	559
Espritz-X	550	553
Espritz-X	564	571
Espritz-N	585	587
PrDOS	591	628
VLXT	592	616
IUPred-S	593	593
IUPred-L	593	593
Espritz-N	594	607
IUPred-L	597	600
IUPred-S	599	600
IUPred-L	603	607
PV2	613	613
VSL2b	614	615
PV2	615	615
VLXT	626	633
PrDOS	655	672
VSL2b	660	666
Espritz-N	660	663
PV2	662	662
PV2	664	667
VLXT	678	681
VSL2b	682	686
PV2	685	688
PrDOS	710	725
VSL2b	713	716
PV2	716	717
PrDOS	731	733
VLXT	736	772
PV2	749	753
Espritz-N	757	771
VSL2b	760	771
IUPred-L	760	760
PV2	762	768
IUPred-L	765	766
IUPred-S	766	767
PV2	770	770
Espritz-N	784	789
PrDOS	785	884
VSL2b	786	790
PV2	786	787
PV2	789	791
IUPred-L	794	794
PV2	814	873
VSL2b	818	874
Espritz-N	818	837

VLXT	820	850
IUPred-S	823	843
IUPred-L	823	841
Espritz-X	826	840
VLXT	854	876
Espritz-N	862	876
IUPred-L	865	865
IUPred-S	867	871
IUPred-L	867	869
IUPred-L	872	872
IUPred-S	874	874
VLXT	925	925
VSL2b	944	945
PV2	961	1002
VSL2b	966	1002
VLXT	977	994
PrDOS	981	1002
Espritz-N	982	990
Espritz-X	983	1002
IUPred-S	988	991
IUPred-L	989	990
IUPred-S	996	1002
VLXT	1001	1001

Table S2. TFs used in Y2H assay

Arabidopsis TF library number (1)	TF family	Accession	Gene name	Interaction with SMAX1 orSMXL7	References
DEST-U18-D06	ABI3-VP1	AT3G24650.1	ABI3	No	Seed dormancy(2, 3)
DEST-U13-F01	ALFIN-like	AT2G02470.2	AL6	No	Root hair development(4)
DEST-U17-C04	AP2-EREBP	AT5G10510.1	AIL6	No	*rac*-GR24 responsive Differently Accessible Region (DAR)(5)
DEST-U15-G10	AP2-EREBP	AT1G24590.1	BOL	SMAX1	Germination and embryo morphogenesis (2)
DEST-U20-C11	AP2-EREBP	AT1G12980.1	DRN	SMAX1	Germination and embryo morphogenesis (2)
DEST-U04-A08	AP2-EREBP	AT3G23240.1	ERF1	No	Photomorphogenesis(6)
DEST-U05-A09	AP2-EREBP	AT4G34410.1	ERF109	No	$GR24^{4DO}$-responsive gene(7)
DEST-U07-F04	AP2-EREBP	AT1G28370.1	ERF11	No	*rac*-GR24 responsive gene(5)
DEST-U16-H12	AP2-EREBP	AT5G25810.1	ERF40	No	*rac*-GR24 responsive DAR(5)
DEST-U05-E05	AP2-EREBP	AT5G65130.1	ERF57	No	*rac*-GR24 responsive gene(5)
DEST-U17-D07	AP2-EREBP	AT4G17490.1	ERF6	No	*rac*-GR24 responsive DAR(5)
DEST-U01-H06	AP2-EREBP	AT1G64380.1	ERF61	SMAX1 and SMXL7	SMXL6-targeted and *rac*-GR24-responsive gene(5, 7)
DEST-U05-C06	AP2-EREBP	AT3G16770.1	ERF72	No	SMXL6-targeted(7)
DEST-U05-F07	AP2-EREBP	AT5G44210.1	ERF9	No	SMXL6-targeted(7)
DEST-U03-E05	AP2-EREBP	AT5G25190.1	ESE3	No	SMXL6-targeted(7)
DEST-U18-D08	AP2-EREBP	AT3G20840.1	PLT1	No	Germination and embryo morphogenesis (2)
DEST-U05-E01	AP2-EREBP	AT1G51190.1	PLT2	No	Germination and embryo morphogenesis (2)
DEST-U19-G05	ARF	AT1G59750.1	ARF1	No	*rac*-GR24 responsive gene(5)
DEST-U07-B08	ARF	AT3G61830.1	ARF18	No	*rac*-GR24 responsive gene(5)
DEST-U05-H03	AUX/IAA	AT3G15540.1	IAA19	No	SMXL6-targeted(7)
DEST-U18-C01	AUX/IAA	AT4G32280.1	IAA29	No	Cell elongation regulation(8)
DEST-U04-F12	AUX/IAA	AT5G43700.1	IAA4	No	*rac*-GR24 responsive gene(5)
DEST-U02-D06	AUX/IAA	AT1G15580.1	IAA5	No	SMXL6-targeted(7)
DEST-U04-F08	BES1	AT3G50750.1	BEH1	No	Arabidopsis homolog of OsBES1(9), Photomorphogenesis and seed germination(10)
DEST-U17-F04	BES1	AT4G36780.2	BEH2	No	Arabidopsis homolog of OsBES1(9), Photomorphogenesis and seed germination(10)
DEST-U06-F06	BES1	AT4G18890.1	BEH3	No	Arabidopsis homolog of OsBES1(9)
DEST-U16-H10	BES1	AT1G19350.1	BES1	No	Arabidopsis homolog of OsBES1(9), Photomorphogenesis and seed germination(10)
DEST-U10-C09	BES1	AT1G75080.1	BZR1	No	Photomorphogenesis and seed germination(10)
DEST-U15-G04	bHLH	AT1G68810.1	ABS5/T5L1	SMAX1	Germination(2)
DEST-U12-E12	bHLH	AT2G28160.1	FIT	No	GR244DO-responsive and targeted by SMXL6(7)
DEST-U01-D07	bHLH	AT5G67060.1	HEC1	No	GR244DO-responsive and targeted by SMXL6(7)
DEST-U02-F12	bHLH	AT4G30180.1	HLH4	No	Cell elongation and anthocyanin accumulation(11)
DEST-U19-A07	bHLH	AT2G27230.1	LHW	No	Germination and Seedling development(2)
DEST-U19-B01	bHLH	AT1G32640.1	MYC2	No	Photomorphogenesis(12)
DEST-U13-A07	bHLH	AT5G46760.1	MYC3	No	Seed germination regulation(13), *rac*-GR24-responsive gene(5)
DEST-U13-A05	bHLH	AT4G17880.1	MYC4	No	Seed germination regulation(13)
DEST-U01-C06	bHLH	AT2G20180.2	PIF1	No	Photomorphogenesis(14)
DEST-U15-F08	bHLH	AT2G43010.1	PIF4	No	Photomorphogenesis(10, 15), thermomorphogenesis(16)
DEST-U20-E03	bHLH	AT3G59060.2	PIF5	No	Photomorphogenesis(14)

DEST-U09-H06	bHLH	AT5G61270.1	PIF7	No	Photomorphogenesis and thermomorphogenesis(17)
DEST-U07-A04	bHLH	AT4G00050.1	PIF8	No	Photomorphogenesis(18), potential genetic interaction downstream of PhyB(16)
DEST-U04-G09	bHLH	AT1G66470.1	RHD6	No	Root hair development(4)
DEST-U09-H05	bHLH	AT5G37800.1	RSL1	No	Root hair development(4)
DEST-U01-C01	bHLH	AT1G09530.1	PIF3	SMAX1	Photomorphogenesis(10, 15), thermomorphogenesis(19)
DEST-U06-H12	bHLH	AT4G36930.1	SPT	SMAX1	Seed dormancy(2)
DEST-U03-F05	bHLH	AT3G25710.1	TMO5	SMAX1	Seed development(2)
DEST-U06-B10	bZIP	AT1G49720.1	ABF1	No	Germination(3)
DEST-U06-B05	bZIP	AT1G45249.1	ABF2	No	Seed dormancy(2, 3)
DEST-U18-E06	bZIP	AT4G34000.1	ABF3	No	Germination(3)
DEST-U02-H03	bZIP	AT3G19290.1	ABF4	No	Germination(3)
DEST-U06-B06	bZIP	AT2G36270.1	ABI5	No	Seed dormancy(2, 3)
DEST-U09-F02	bZIP	AT4G01120.1	bZIP54/GBF2	No	Seed development(2)
DEST-U03-D06	bZIP	AT3G44460.1	bZIP67	No	Seed dormancy(2, 3)
DEST-U16-B08	bZIP	AT5G11260.1	HY5	No	Photomorphogenesis(15), seed germination(10)
DEST-U02-B02	bZIP	AT3G17609.4	HYH	No	Photomorphogenesis(15)
DEST-U12-F01	bZIP	AT1G22070.1	TGA3/bZIP22	SMAX1	Seed dormancy regulation(3)
DEST-U01-F06	C2C2-CO-like	AT2G47890.1	BBX11	No	Photomorphogenesis, thermomorphogenesis, and flowering (10, 20)
DEST-U08-A07	C2C2-CO-like	AT2G24790.1	BBX4	No	Photomorphogenesis, flowering(10, 20)
DEST-U11-D09	C2C2-DOF	AT3G61850.1	DAG1	No	Germination regulation(3)
DEST-U05-F02	C2C2-YABBY	AT2G45190.1	YAB1/FIL	No	Germination and embryo morphogenesis (2)
DEST-U01-E05	C2C2-YABBY	AT1G08465.1	YAB2	No	Germination and embryo morphogenesis (2)
DEST-U04-B10	C2C2-YABBY	AT4G00180.1	YAB3	No	Germination and embryo morphogenesis (2)
DEST-U07-G05	C2H2	AT2G34500.1	FZF	No	*rac*-GR24 responsive DAR(5)
DEST-U06-G09	C2H2	AT1G10480.1	ZFP5	No	Root hair development(4)
DEST-U17-B02	EIL	AT2G27050.1	EIL1	No	Photomorphogenesis(6), Root hair development(4)
DEST-U19-B12	EIL	AT3G20770.1	EIN3	No	Photomorphogenesis(6), Root hair development(4)
DEST-U06-C01	G2-like	AT5G16560.1	KAN1	No	Germination and embryo morphogenesis(2), Leaf development(21)
DEST-U16-B05	G2-like	AT1G32240.1	KAN2	No	Germination and embryo morphogenesis(2), leaf development(22)
DEST-U06-H02	G2-like	AT5G42630.1	KAN4	No	Germination and embryo morphogenesis(2)
DEST-U17-B04	GNAT	AT4G37580.1	COP3/HLS1	No	Thermomorphogenesis(23)
DEST-U04-H01	GRAS	AT2G01570.1	RGA	SMAX1	Photomorphogenesis and seed germination(10, 24), seed dormancy(2)
DEST-U12-E08	GRAS	AT1G66350.1	RGL1	SMAX1 and SMXL7	Seed dormancy and germination, photomorphogenesis(10)
DEST-U04-C04	GRAS	AT1G14920.1	GAI	SMAX1 and SMXL7	Photomorphogenesis and seed germination(10, 24)
DEST-U07-F03	GRAS	AT5G17490.1	RGL3	SMAX1 and SMXL7	Photomorphogenesis and seed germination(10, 24), seed dormancy(2)
DEST-U16-C02	GRF	AT2G22840.1	GRF1	No	Arabidopsis homolog of OsGRF4(25)
DEST-U12-F05	GRF	AT4G37740.1	GRF2	No	Arabidopsis homolog of OsGRF4(25)
DEST-U11-A10	GRF	AT2G36400.1	GRF3	No	Arabidopsis homolog of OsGRF4(25)
DEST-U17-B10	GRF	AT3G52910.1	GRF4	No	Arabidopsis homolog of OsGRF4(25)
DEST-U16-H03	GRF	AT5G53660.1	GRF7	No	Arabidopsis homolog of OsGRF4(25)
DEST-U16-C04	GRF	AT2G45480.1	GRF9	No	Arabidopsis homolog of OsGRF4(25)
DEST-U04-C06	HB	AT3G01470.1	ATHB1	No	Hypocotyl elongation(26), *rac*-GR24-responsive gene(5)

DEST-U03-E09	HB	AT4G16780.1	ATHB2/HAT4	No	Germination and embryo morphogenesis(2)
DEST-U01-H02	HB	AT2G44910.1	ATHB4	SMAX1	Germination and embryo morphogenesis(2)
DEST-U18-D04	HB	AT2G34710.1	ATHB14/PHB	SMAX1	Germination and embryo morphogenesis(2)
DEST-U19-G03	HB	AT1G52150.1	ATHB15/CNA	SMAX1 and SMXL7	Germination and embryo morphogenesis(2)
DEST-U19-G04	HB	AT1G30490.1	ATHB9/PHV	SMAX1 and SMXL7	Germination and embryo morphogenesis(2)
DEST-U19-G09	HB	AT1G79840.1	GL2	No	Root hair development(4)
DEST-U04-G11	HB	AT3G60390.1	HAT3	No	Germination and embryo morphogenesis(2)
DEST-U14-A07	HB	AT4G04890.1	PDF2	SMAX1 and SMXL7	Germination and embryo morphogenesis(2)
DEST-U01-D09	HB	AT5G59340.1	WOX2	No	Germination and embryo morphogenesis(2)
DEST-U01-D10	HB	AT5G45980.1	WOX8	SMAX1 and SMXL7	Germination and embryo morphogenesis(2)
DEST-U01-D10	HB	AT2G33880.1	WOX9	SMAX1 and SMXL7	Germination and embryo morphogenesis(2)
DEST-U14-D08	HSF	AT3G22830.1	HSFA6B	No	$GR24^{4DO}$-responsive gene(7)
DEST-U16-B06	HSF	AT1G46264.1	HSFB4	No	*rac*-GR24 responsive DAR(5)
DEST-U19-E12	JUMONJI	AT1G09060.1	JMJ24	No	Arabidopsis homolog of OsGRF4(25)
DEST-U09-G11	JUMONJI	AT3G20810.1	JMJ30	No	Post-germination regulation(3)
DEST-U02-F05	LOB/AS2	AT2G42430.1	LBD16	No	$GR24^{4DO}$-responsive gene(7)
DEST-U03-F04	MYB	AT5G45420.1	maMYB	No	Root hair development(4)
DEST-U06-B01	MYB	AT3G23250.1	MYB15	No	*rac*-GR24 responsive gene(5)
DEST-U17-F01	MYB	AT1G56650.1	PAP1	No	$GR24^{4DO}$-responsive gene(7)
DEST-U12-H05	MYB-related	AT5G17300.1	RVE1	No	Seed dormancy and germination(3, 27)
DEST-U05-H09	MYB-related	AT1G01520.1	RVE3	No	Seed dormancy, germination and embryo morphogenesis(2)
DEST-U06-F12	NAC	AT3G15170.1	CUC1	No	Germination and embryo morphogenesis(2), leaf development (21)
DEST-U06-C02	NAC	AT5G53950.1	CUC2	No	Germination and embryo morphogenesis(2), leaf development (21)
DEST-U13-B03	NAC	AT1G76420.1	CUC3	No	Germination and embryo morphogenesis(2)
DEST-U03-G02	NAC	AT3G49530.1	NAC062	No	*rac*-GR24 responsive gene(5)
DEST-U06-G05	NAC	AT5G62380.1	NAC101	No	SMXL6-targeted(7)
DEST-U14-C09	Orphans	AT2G21320.1	BBX18	No	Photomorphogenesis(10)
DEST-U05-A07	Orphans	AT4G38960.1	BBX19	No	Photomorphogenesis and flowering(10, 20)
DEST-U01-G04	Orphans	AT4G39070.1	BBX20	No	Photomorphogenesis(10, 20)
DEST-U18-H01	Orphans	AT1G75540.1	BBX21	No	Seed germination(10, 20)
DEST-U10-D03	Orphans	AT1G78600.1	BBX22	No	Photomorphogenesis(10, 20)
DEST-U03-B06	Orphans	AT4G10240.1	BBX23	No	Photomorphogenesis and thermomorphogenesis(10, 20)
DEST-U02-H01	Orphans	AT1G06040.1	BBX24	No	Photomorphogenesis(10, 20)
DEST-U02-H09	Orphans	AT2G31380.1	BBX25	No	Photomorphogenesis(10, 20)
DEST-U12-D10	Orphans	AT4G27310.1	BBX28	No	Photomorphogenesis(10, 20)
DEST-U17-E01	Orphans	AT5G54470.1	BBX29	No	Photomorphogenesis(10, 20)
DEST-U08-E06	Orphans	AT4G15248.1	BBX30	SMAX1	Photomorphogenesis(10, 20)
DEST-U14-B09	Orphans	AT3G21890.1	BBX31	No	Photomorphogenesis(10, 20)
DEST-U20-G12	Orphans	AT3G23150.1	ETR2	No	$GR24^{4DO}$-responsive gene(7)
DEST-U19-F06	Orphans	AT4G21430.1	JMJ28	No	Arabidopsis homolog of OsGRF4(25)
DEST-U14-G05	Orphans	AT1G74890.1	ARR15	No	Seed development(14)
DEST-U14-G06	Orphans	AT2G41310.1	ARR8	No	SMXL6-targeted(7)
DEST-U19-A05	SBP	AT2G47070.1	SPL1	No	*Arabidopsis* homolog of OsIPA1(28)

DEST-U06-H04	SBP	AT5G43270.1	SPL2	No	*Arabidopsis* homolog of OsIPA1(28)
DEST-U03-C12	SBP	AT1G53160.1	SPL4	No	*Arabidopsis* homolog of OsIPA1(28)
DEST-U04-E09	SBP	AT3G15270.1	SPL5	No	*Arabidopsis* homolog of OsIPA1(28)
DEST-U04-E08	SBP	AT1G69170.1	SPL6	No	*Arabidopsis* homolog of OsIPA1(28)
DEST-U19-G10	SBP	AT5G18830.1	SPL7	No	*Arabidopsis* homolog of OsIPA1(28)
DEST-U11-A06	SBP	AT1G02065.1	SPL8	No	*Arabidopsis* homolog of OsIPA1(28)
DEST-U04-H02	SBP	AT2G42200.1	SPL9	No	*Arabidopsis* homolog of OsIPA1(28)
DEST-U03-H07	SBP	AT1G27370.1	SPL10	No	*Arabidopsis* homolog of OsIPA1(28)
DEST-U03-C08	SBP	AT1G27360.1	SPL11	No	*Arabidopsis* homolog of OsIPA1(28)
DEST-U19-E05	SBP	AT3G60030.1	SPL12	No	*Arabidopsis* homolog of OsIPA1(28)
DEST-U11-G04	SBP	AT5G50570.1	SPL13	No	*Arabidopsis* homolog of OsIPA1(28)
DEST-U21-A02	SBP	AT1G20980.1	SPL14	No	*Arabidopsis* homolog of OsIPA1(28)
DEST-U02-E03	SBP	AT3G57920.1	SPL15	No	*Arabidopsis* homolog of OsIPA1(28)
DEST-U17-B01	SIGMA70-like	AT3G53920.1	SIG3	No	Anterograde signals downstream of PIFs to regulate photoresponses(29)
DEST-U06-B07	TCP	AT5G60970.1	TCP5	SMAX1	Hypocotyl elongation(30, 31), member of TCP family
DEST-U20-B12	TCP	AT5G23280.1	TCP7	SMAX1 and SMXL7	Hypocotyl elongation and leaf petiole(32), member of TCP family
DEST-U07-C02	TCP	AT1G58100.1	TCP8	SMAX1 and SMXL7	Hypocotyl elongation and leaf petiole(32), member of TCP family
DEST-U07-F11	TCP	AT2G45680.1	TCP9	SMAX1 and SMXL7	Member of TCP family, leaf development(33)
DEST-U07-G07	TCP	AT2G31070.1	TCP10	SMAX1 and SMXL7	Hypocotyl and leaf(34, 35), member of TCP family
DEST-U17-E11	TCP	AT1G68800.2	TCP12/BRC2	No	Branching(36, 37), member of TCP family
DEST-U09-E02	TCP	AT3G02150.2	TCP13	SMAX1 and SMXL7	Hypocotyl and leaf(30, 38), member of TCP family
DEST-U04-A09	TCP	AT3G47620.1	TCP14	SMAX1 and SMXL7	Hypocotyl and leaf petiole(32, 39), member of TCP family
DEST-U15-D12	TCP	AT3G45150.1	TCP16	SMAX1 and SMXL7	Leaf development(40), member of TCP family
DEST-U01-E03	TCP	AT5G08070.1	TCP17	SMAX1	Hypocotyl elongation(30), member of TCP family
DEST-U15-E01	TCP	AT3G18550.1	TCP18/BRC1	SMAX1 and SMXL7	Branching(41–43), member of TCP family
DEST-U18-C02	TCP	AT5G51910.1	TCP19	SMAX1 and SMXL7	Member of TCP family, leaf senescence(33)
DEST-U20-D12	TCP	AT5G08330.1	TCP21	SMAX1	Hypocotyl elongation and leaf petiole(32), member of TCP family
DEST-U14-D09	TCP	AT1G31210.1	TCP24	No	Hypocotyl elongation and leaf development(34, 35), member of TCP family
DEST-U05-C04	TCP	AT2G37000.1	TCP (AT2G37000)	SMAX1	member of TCP family
DEST-U01-E01	TCP	AT5G41030.1	TCP (AT5G41030)	No	member of TCP family
DEST-U19-B11	WRKY	AT5G56270.1	WRKY2	No	Germination and embryo morphogenesis(2)
DEST-U14-A02	WRKY	AT2G38470.1	WRKY33	No	Phosphate-deficiency induced root architecture regulation(44)
DEST-U09-C09	WRKY	AT4G04450.1	WRKY42	No	*rac*-GR24 responsive DAR(5)
DEST-U05-D04	WRKY	AT5G13080.1	WRKY75	No	Root hair development(4)

Table S3. Primers

I. Primers for cloning of pGWBcitr-SMAX1pro and pGWBcitr-SMXL7pro
(Backbone plasmid, lowercase; Insert, uppercase)

Name	5'-3' sequence	Note
Citrine cassette-5F	acgccgttgatgtggacgccgTTACTCTTCTTCTTGATCA	ApaI site of pGWB501
Citrine cassette-3R	gttgaaggagccactcagccGAAAGGGGTTTAGGGTTTAA	SacII site of pGWB501
pGWB-SMXL7pro-5F	ttgcatgcctgcaggtcgactCTATTTTATTTATGTGACAGTTT	XbaI site of pGWB501
pGWB-SMXL7pro-3R	tttttgtacaaacttgttgataactCTAGCGTCGCCGGTTTAGTT	XbaI site of pGWB501
pGWB-SMAX1pro-5F	ttgcatgcctgcaggtcgactAAAAGTAGATTTATTTTGTA	XbaI site of pGWB501
pGWB-SMAX1pro-3R	tttttgtacaaacttgttgataactCGTTCTTCGTTTACTTCCAC	XbaI site of pGWB501

II. Primers for cloning Gateway entry clones
(attB, lowercase; overlapped flanking sequences, underlined lowercase; start codons, red; stop codons, blue)

BP221-SMAX1-5F	ggggacaagtttgtacaaaaaagcaggctcgATGAGAGCTGGTTTAAGTAC	Amplification of SMAX1
BP221-SMXL7-5F	ggggacaagtttgtacaaaaaagcaggctccATGCCGACACCAGTAACC	Amplification of SMXL7
OE-SMAX1-FLAG-3R	tccttgtaatcgccggatccgccTACTGCCAAAGTAATAGTTGTC	Amplification of SMAX1 with overlapping sequence for FLAG
OE-SMXL7-FLAG-3R	tccttgtaatcgccggatccgccGATCACTTCGACTCTCG	Amplification of SMXL7 with overlapping sequence for FLAG
GGSG-FLAG-5F	GGCGGATCCGGCGATTACAA	Amplification of FLAG
BP221-FLAG-3R	ggggaccactttgtacaagaaagctgggtcCTACTTGTCATCATCATCCTTGTAATC	Amplification of FLAG
SMAX1$_{N}$-158-3R	TTTAACGGCGGGACTTGAAAAG	Amplification of SMAX1$_{N}$
OE-SMXLχ71O-5F	gaagcaccgacattaaaGCTACAATTGAACAGTCGTTG	Amplification of SMAX1$_{D1M}$ with overlapping sequence for SMXL7$_{N}$
OE-SMXLχO17-3R	gagagtagttccctgagagaCTTAAACAAATCAATGTCTAACG	Amplification of SMAX1$_{D1M}$ with overlapping sequence for SMXL7$_{D2}$
SMAX1$_{D2}$-611-5F	AAGCTGTTGAAGGGAATGAC	Amplification of SMAX1$_{D2}$
SMXL7$_{N}$-174-3R	TTTAATGTCGGTGCTTCTAAAC	Amplification of SMXL7$_{N}$
OE-SMXLχ17O-5F	cttttcaagtcccgccgttaaaCTCGACGTGCTTCATCCTCC	Amplification of SMXL7$_{D1M}$ with overlapping sequence for SMAX1$_{N}$
OE-SMXLχO71-3R	gtcattcccttcaacagcttCTTGAAATCTTTGCAGTATCTC	Amplification of SMXL7$_{D1M}$ with overlapping sequence for SMAX1$_{D2}$
SMXL7$_{D2}$-630-5F	TCTCTCAGGGAACTACTCTCTC	Amplification of SMXL7$_{D2}$
BP221-SV40 NLS type1-5F	ggggacaagtttgtacaaaaaagcaggctcgATGCCCAAGAAGAAGCGTAAGG	Amplification of SV40 NLS-fused SMAX1$_{N}$ or SMXL7$_{N}$
BP221-SV40NLS type2-5F	ggggacaagtttgtacaaaaaagcaggctcaATGCCAAAAAAGAAGAGAAAGG	Amplification of SV40 NLS-fused SMAX1ΔN or SMXL7ΔN
OE-NLS-SMAX1ΔN-5F	aaaggtagaagaccccactagtGCTACAATTGAACAGTCG	Amplification of SV40 NLS-fused SMAX1ΔN
OE-NLS-SMXL7ΔN-5F	aaaggtagaagaccccactagtCTCGACGTGCTTCATCCT	Amplification of SV40 NLS-fused SMXL7ΔN
BP221-SRDX-stop-3R	ggggaccactttgtacaagaaagctgggtcTCAAGCGAAACCGAGCCTGAGCT	Amplification of SRDX-fused constructs
SMXL7ΔRGKT-5F	GACAGTCTTGACGATAGATTCGTTGTTGATTACATTGCTGG	For removal of RGKT motif of SMXL7
SMXL7ΔRGKT-3R	GAATCTATCGTCAAGACTGTC	For removal of RGKT motif of SMXL7
OE-NLS-GFP-5F	aaaggtagaagaccccactagtATGGTGAGCAAGGGCGAGGA	Amplification of of NLS-GFP constructs
OE-GFP-FLAG-3R	tccttgtaatcgccggatccgccCTTGTACAGCTCGTCCATGCC	Amplification of GFP with overlapping sequence for FLAG tag
SMAX1$_{N210}$-3R	TTGTACCGACGAAGCGTTCTG	Amplification of SMAX1$_{N210}$
OE-SMXLχ1$_{210}$7O-5F	TCGGTACAAACGTCAAGATCTCGTATTCCTC	Amplification of extended SMXL7$_{D1M}$ with overlapping sequence for SMAX1$_{N210}$

III. Primers for genotyping

smax1-2_LP	GTGGCAACTGTTTAGGCTGAG	Li et al., 2022, paired with LBb1.3 (45)
smax1-2_RP	AAGCTAGCTTTTCAAGTCCCG	Li et al., 2022, paired with LBb1.3 (45)
smxl2-1_RP	CCACTTCAGTGTCGAGCTCTC	paired with LB1
smxl2-1_LP	TTGCTCCCAAGCCTAATCAAAAC	paired with LB1
smxl6-4_LP	AGCCAGAGAAAGACTCGAACC	Wang et al., 2015, paired with LBb1.3 (40)
smxl6-4_RP	TCCGAAATTAAGCTCGATGTG	Wang et al., 2015, paired with LBb1.3 (40)
smxl7-3_LP	GATCAAGAAACGAACGCTGAG	Wang et al., 2015, paired with WiscDsLox-LB (40)
smxl7-3_RP	CGTATTAGCCTCTCGGATTCC	Wang et al., 2015, paired with WiscDsLox-LB (40)
smxl8-1_LP	GAATCACAAATTCTGCATGGC	Wang et al., 2015, paired with LBb1.3 (40)
smxl8-1_RP	CTGACGAAGCTCCACTTTCAC	Wang et al., 2015, paired with LBb1.3 (40)
max3-9-F	GGTCACTTGCAACGCTGAAG	*max3-9* 102bp; MAX3 75bp
max3-9-R	GAATTAAAAGATTATTTACCACAAAATGTGAAGTTGCT	*max3-9* 102bp; MAX3 75bp
SAIL_LB1	GCCTTTTCAGAAATGGATAAATAGCCTTGCTTCC	
SALK_LBb1.3	ATTTTGCCGATTTCGGAAC	
WiscDsLox-LB-p745	AACGTCCGCAATGTGTTATTAAGTTGTC	

IV. Primers for qRT-PCR

CACS/AP2M-qRT-5F	ACTCAGGAAGGTGTACGGTCA	Reference gene
CACS/AP2M-qRT-3R	TGCATTTGGAACAGGTTTGT	Reference gene
SV40NLS-qRT-5F	CGTAAGGTCGAGGACCCCATG	For SMAX1$_{N158}$-SRDX, SMAX1$_{N210}$-SRDX, and SMXL7$_{N174}$-SRDX
NLS-SMAX1$_{N}$-qRT-3R	GGTTTAAAACGGTGGCAGCC	For SMAX1$_{N158}$-SRDX and SMAX1$_{N210}$-SRDX
SMAX1-qRT-5F	CGGGTCGGGTTATTCGTGAG	Park et al. 2022 (16)
SMAX1-qRT-3R	GGGTCTGAAGCAACCCATCT	Park et al. 2022 (16)
SMXLχ177-qRT-5F	CTAGCTTTTCAAGTCCCGC	For SMXLχ177 qRT-PCR
SMXLχ177-qRT-3R	GAATCTAACCCGACCCGAGTC	For SMXLχ177
KUF1-qRT-5F	AACCCGTCAGTCCGGTTTATG	
KUF1-qRT-3R	AACGACGGATGACGGTAAAGA	
DLK2-qRT-5F	GCTGCTTCTCCAAGGTATATAAACAGTG	
DLK2-qRT-3R	GAAATCAACCGCCCAAGCT	
ERF61-qRT-5F	CGACGGGAAGATCCAATCCA	Chang et al., 2024 (45)
ERF61-qRT-5F	CGGCGATGACAAACACGAAG	Chang et al., 2024 (45)
IAA29-qRT-5F	ATGGATGGTGTGGCAATA	Chang et al., 2024 (45)
IAA29-qRT-3R	ATCTTCTCTGTCGCAATCT	Chang et al., 2024 (45)
BBX20/STH7-qRT-5F	CATCTCCGGTTCTCTCTCACTTCT	Sepulveda et al., 2020 (46)
BBX20/STH7-qRT-3R	CATTCTCTGCATAGTATTGCTCTGTC	Sepulveda et al., 2020 (46)
SMXL7-qRT-5F	CCAGTGATTGTCATGTTCTT	Wang et al., 2015 (42)
SMXL7-qRT-3R	TCCTCCTGTCTTCTCTTATTG	Wang et al., 2015 (42)
NLS-SMXL7$_{N}$-qRT-3R	CTGAAACGGCGTGAAGTGAC	Reverse primer for analyzing SMXL7$_{N174}$-SRDX
SMXLχ711-qRT-5F	GGTTACAACAGAACGCTTCG	
SMXLχ711-qRT-3R	GATCTCACGAATAACCCGAC	

BRC1-qRT-5F	CCAGTGATTAACCACCATC	Wang et al., 2015 (42)
BRC1-qRT-3R	GCCGAAGGAGTAATGAAG	Wang et al., 2015 (42)
TCP1-qRT-5F	ATCAGAGTTCTAGCAGCAA	Wang et al., 2020 (7)
TCP1-qRT-3R	GAGTCTTGAATCCAAACTTTAC	
PAP1-qRT-5F	GAAGCGACGACAACAGAA	Lee et al., 2024 (47)
PAP1-qRT-3R	TGAAAGCAAACCTATACACAAAC	Lee et al., 2024 (47)

SI References

1. J. L. Pruneda-Paz, *et al.*, A genome-scale resource for the functional characterization of Arabidopsis transcription factors. *Cell Rep.* **8**, 622–632 (2014).

2. S. Verma, V. P. S. Attuluri, H. S. Robert, Transcriptional control of Arabidopsis seed development. *Planta* **255**, 90 (2022).

3. F. Ali, G. Qanmber, F. Li, Z. Wang, Updated role of ABA in seed maturation, dormancy, and germination. *J. Advert. Res.* **35**, 199–214 (2022).

4. M. Shibata, K. Sugimoto, A gene regulatory network for root hair development. *J. Plant Res.* **132**, 301–309 (2019).

5. J. L. Humphreys, C. Beveridge, M. Tanurdzic, Strigolactone-dependent gene regulation requires chromatin remodeling. *bioRxiv* 2023.02.25.529999 (2023).

6. H. Shi, *et al.*, Genome-wide regulation of light-controlled seedling morphogenesis by three families of transcription factors. *Proc. Natl. Acad. Sci. U. S. A.* **115**, 6482–6487 (2018).

7. L. Wang, *et al.*, Transcriptional regulation of strigolactone signalling in Arabidopsis. *Nature* **583**, 277–281 (2020).

8. O. Pucciariello, *et al.*, Rewiring of auxin signaling under persistent shade. *Proc. Natl. Acad. Sci. U. S. A.* **115**, 5612–5617 (2018).

9. J. Hu, Y. Ji, X. Hu, S. Sun, X. Wang, BES1 Functions as the Co-regulator of D53-like SMXLs to Inhibit BRC1 Expression in Strigolactone-Regulated Shoot Branching in Arabidopsis. *Plant Commun* **1**, 100014 (2020).

10. J. Cao, *et al.*, Multi-layered roles of BBX proteins in plant growth and development. *Stress Biol* **3**, 1 (2023).

11. Q. Hou, *et al.*, Overexpression of HLH4 Inhibits Cell Elongation and Anthocyanin Biosynthesis in Arabidopsis thaliana. *Cells* **11** (2022).

12. Y. Jiao, O. S. Lau, X. W. Deng, Light-regulated transcriptional networks in higher plants. *Nat. Rev. Genet.* **8**, 217–230 (2007).

13. L. Ju, *et al.*, JAZ proteins modulate seed germination through interaction with ABI5 in bread wheat and Arabidopsis. *New Phytol.* **223**, 246–260 (2019).

14. P. Wang, *et al.*, Photomorphogenesis in plants: The central role of phytochrome interacting factors (PIFs). *Environ. Exp. Bot.* **194**, 104704 (2022).

15. K.-P. Jia, Q. Luo, S.-B. He, X.-D. Lu, H.-Q. Yang, Strigolactone-regulated hypocotyl elongation is dependent on cryptochrome and phytochrome signaling pathways in Arabidopsis. *Mol. Plant* **7**, 528–540 (2014).

16. Y.-J. Park, J. Y. Kim, C.-M. Park, SMAX1 potentiates phytochrome B-mediated hypocotyl thermomorphogenesis. *Plant Cell* **34**, 2671–2687 (2022).

17. Y. Burko, *et al.*, PIF7 is a master regulator of thermomorphogenesis in shade. *Nat. Commun.* **13**, 4942 (2022).

18. J. Ding, B. Zhang, Y. Li, D. André, O. Nilsson, Phytochrome B and PHYTOCHROME INTERACTING FACTOR8 modulate seasonal growth in trees. *New Phytol.* **232**, 2339–2352 (2021).

19. Y. Bian, *et al.*, PIFs- and COP1-HY5-mediated temperature signaling in higher plants. *Stress Biol* **2**, 35 (2022).

20. A. Yadav, N. Ravindran, D. Singh, P. V. Rahul, S. Datta, Role of Arabidopsis BBX proteins in light signaling. *J. Plant Biochem. Biotechnol.* **29**, 623–635 (2020).

21. S. Ali, N. Khan, L. Xie, Molecular and Hormonal Regulation of Leaf Morphogenesis in Arabidopsis. *Int. J. Mol. Sci.* **21** (2020).

22. M. A. Romanova, V. V. Domashkina, A. I. Maksimova, K. Pawlowski, O. V. Voitsekhovskaja, All together now: Cellular and molecular aspects of leaf development in lycophytes, ferns, and seed plants. *Frontiers in Ecology and Evolution* **11** (2023).

23. H. Jin, Z. Zhu, HOOKLESS1 is a positive regulator in Arabidopsis thermomorphogenesis. *Sci. China Life Sci.* **62**, 423–425 (2019).

24. H. Zhao, Y. Zhang, Y. Zheng, Integration of ABA, GA, and light signaling in seed germination through the regulation of ABI5. *Front. Plant Sci.* **13**, 1000803 (2022).

25. H. Sun, *et al.*, Strigolactone and gibberellin signaling coordinately regulate metabolic adaptations to changes in nitrogen availability in rice. *Mol. Plant* **16**, 588–598 (2023).

26. M. Capella, P. A. Ribone, A. L. Arce, R. L. Chan, Arabidopsis thaliana HomeoBox 1 (AtHB1), a Homedomain-Leucine Zipper I (HD-Zip I) transcription factor, is regulated by PHYTOCHROME-INTERACTING FACTOR 1 to promote hypocotyl elongation. *New Phytol.* **207**, 669–682 (2015).

27. Z. Jiang, G. Xu, Y. Jing, W. Tang, R. Lin, Phytochrome B and REVEILLE1/2-mediated signalling controls seed dormancy and germination in Arabidopsis. *Nat. Commun.* **7**, 12377 (2016).

28. X. Song, *et al.*, IPA1 functions as a downstream transcription factor repressed by D53 in strigolactone signaling in rice. *Cell Res.* **27**, 1128–1141 (2017).

29. Y. Hwang, *et al.*, Anterograde signaling controls plastid transcription via sigma factors separately from nuclear photosynthesis genes. *Nat. Commun.* **13**, 7440 (2022).

30. Y. Zhou, *et al.*, TCP Transcription Factors Associate with PHYTOCHROME INTERACTING FACTOR 4 and CRYPTOCHROME 1 to Regulate Thermomorphogenesis in Arabidopsis thaliana. *iScience* **15**, 600–610 (2019).

31. X. Han, *et al.*, Arabidopsis Transcription Factor TCP5 Controls Plant Thermomorphogenesis by Positively Regulating PIF4 Activity. *iScience* **15**, 611–622 (2019).

32. W. Zhang, *et al.*, The MPK8-TCP14 pathway promotes seed germination in Arabidopsis. *Plant J.* **100**, 677–692 (2019).

33. S. Danisman, *et al.*, Analysis of functional redundancies within the Arabidopsis TCP transcription factor family. *J. Exp. Bot.* **64**, 5673–5685 (2013).

34. K. R. Challa, P. Aggarwal, U. Nath, Activation of YUCCA5 by the Transcription Factor TCP4 Integrates Developmental and Environmental Signals to Promote Hypocotyl Elongation in Arabidopsis. *Plant Cell* **28**, 2117–2130 (2016).

35. K. R. Challa, M. Rath, U. Nath, The CIN-TCP transcription factors promote commitment to differentiation in Arabidopsis leaf pavement cells via both auxin-dependent and independent pathways. *PLoS Genet.* **15**, e1007988 (2019).

36. M. Muhr, M. Paulat, M. Awwanah, M. Brinkkötter, T. Teichmann, CRISPR/Cas9-mediated knockout of Populus BRANCHED1 and BRANCHED2 orthologs reveals a major function in bud outgrowth control. *Tree Physiol.* **38**, 1588–1597 (2018).

37. P. L. H. Rinne, *et al.*, Long and short photoperiod buds in hybrid aspen share structural development and expression patterns of marker genes. *J. Exp. Bot.* **66**, 6745–6760 (2015).

38. Y.-S. Hur, *et al.*, Arabidopsis transcription factor TCP13 promotes shade avoidance syndrome-like responses by directly targeting a subset of shade-responsive gene promoters. *J. Exp. Bot.* **75**, 241–257 (2024).

39. L. V. Ferrero, V. Gastaldi, F. D. Ariel, I. L. Viola, D. H. Gonzalez, Class I TCP proteins TCP14 and TCP15 are required for elongation and gene expression responses to auxin. *Plant Mol. Biol.* **105**, 147–159 (2021).

40. I. L. Viola, R. Reinheimer, R. Ripoll, N. G. U. Manassero, D. H. Gonzalez, Determinants of the DNA binding specificity of class I and class II TCP transcription factors. *J. Biol. Chem.* **287**, 347–356 (2012).

41. J. A. Aguilar-Martínez, C. Poza-Carrión, P. Cubas, Arabidopsis BRANCHED1 acts as an integrator of branching signals within axillary buds. *Plant Cell* **19**, 458–472 (2007).

42. L. Wang, *et al.*, Strigolactone Signaling in Arabidopsis Regulates Shoot Development by Targeting D53-Like SMXL Repressor Proteins for Ubiquitination and Degradation. *Plant Cell* **27**, 3128–3142 (2015).

43. N. Braun, *et al.*, The pea TCP transcription factor PsBRC1 acts downstream of Strigolactones to control shoot branching. *Plant Physiol.* **158**, 225–238 (2012).

44. N. Shen, S. Hou, G. Tu, W. Lan, Y. Jing, Transcription Factor WRKY33 Mediates the Phosphate Deficiency-Induced Remodeling of Root Architecture by Modulating Iron Homeostasis in Arabidopsis Roots. *Int. J. Mol. Sci.* **22** (2021).

45. W. Chang, *et al.*, Non-transcriptional regulatory activity of SMAX1 and SMXL2 mediates karrikin-regulated seedling response to red light in Arabidopsis. *Mol. Plant* **17**, 1054–1072 (2024).

46. C. Sepulveda, *et al.*, KARRIKIN UP-REGULATED F-BOX 1 (KUF1) imposes negative feedback regulation of karrikin and KAI2 ligand metabolism in *Arabidopsis thaliana*. *Proc. Natl. Acad. Sci. U. S. A.* **119**, e2112820119 (2022).

47. S.-H. Lee, *et al.*, Transcription factors BZR1 and PAP1 cooperate to promote anthocyanin biosynthesis in Arabidopsis shoots. *Plant Cell* **36**, 3654–3673 (2024).

45. Li, Q. et al. The strigolactone receptor D14 targets SMAX1 for degradation in response to GR24 treatment and osmotic stress. Plant Commun 3, 100303 (2022).
